# Contralateral breast cancer after radiotherapy and hormone therapy in two cohorts of US breast cancer survivors

**DOI:** 10.1038/s41416-025-03240-w

**Published:** 2025-11-14

**Authors:** Lene H. S. Veiga, Gretchen L. Gierach, Susan A. Smith, Rebecca M. Howell, Matthew M. Mille, Monjoy Saha, Rochelle E. Curtis, Cody Ramin, Clara Bodelon, Heather Spencer Feigelson, Erin J. Aiello Bowles, Diana S. M. Buist, Sheila Weinmann, Jacqueline B. Vo, Choonsik Lee, Amy Berrington de Gonzalez

**Affiliations:** 1 Division of Cancer Epidemiology and Genetics, National Cancer Institute, Bethesda, MD USA; 2 Department of Radiation Physics, The University of Texas MD Anderson Cancer Center, Houston, TX USA; 3 Samuel Oschin Comprehensive Cancer Institute, Cedars-Sinai Medical Center, Los Angeles, CA USA; 4 Department of Population Science, American Cancer Society, Atlanta, GA USA; 5 Bernard J. Tyson Kaiser Permanente School of Medicine, Pasadena, CA USA; 6 Institute for Health Research, Kaiser Permanente, Denver, CO USA; 7 Kaiser Permanente Washington Health Research Institute, Kaiser Permanente Washington, Seattle, WA USA; 8 Data-driven Strategies for Medicine & Biotechnology, Mercer Island, WA USA; 9 University of Washington, Department of Health Systems and Population, School of Public Health, Seattle, WA USA; 10 Kaiser Permanente Center for Health Research, Portland, OR USA; 11 Division of Genetics & Epidemiology, The Institute of Cancer Research, London, UK

**Keywords:** Breast cancer, Cancer epidemiology

## Abstract

**Background:**

Radiotherapy increases contralateral breast cancer risk, while hormone therapy reduces it; their combined effects are unclear.

**Methods:**

Data from two US retrospective cohort studies of 5-year breast cancer survivors (stage I-III, ages 20–84), Kaiser Permanente (KP, 1990–2012) and SEER (1990–2013), were analysed. Contralateral breast radiation doses were estimated for the KP cohort. Multivariable Poisson regression estimated relative risks (RRs) and excess relative risks per Gray (ERR/Gy), stratified by hormone therapy use.

**Results:**

KP cohort (*n* = 9053) included 353 contralateral breast cancer cases (73% ER+); SEER cohort (*n* = 244,834) included 10,470 cases (72% ER+). Among women with ER+ first breast cancer, radiotherapy increased the risk of ER+ contralateral breast cancer in non-users of hormone therapy (KP RR = 2.2, 95%CI:1.20–4.14; SEER RR = 1.12, 1.04–1.21), but not in users (KP RR = 0.88, 0.61–1.26; SEER RR = 1.03, 0.94–1.12). In KP, higher radiation dose increased risk of ER+ contralateral breast cancer among non-users (ERR/Gy=1.39, 95%CI:0.33,3.66), but not among users (ERR/Gy= –0.13, –0.36,0.23). Radiotherapy also increased risk of ER– contralateral breast cancer (KP RR = 1.85, 95%CI: 0.95–3.59; SEER RR = 1.12, 1.01–1.23), especially in younger exposed women (SEER RR = 1.31, 1.02-1.69 for age <40 vs 40+ years). Additionally, the risk increased linearly with radiation dose to the contralateral breast (ERR/Gy=0.87, 0.04,2.72).

**Conclusions:**

Radiotherapy increased contralateral breast cancer risk, but hormone therapy appeared to mitigate this risk for ER+ cases. These findings have important implications for individuals exposed to chest radiation.

## Introduction

Radiotherapy is an effective breast cancer treatment that reduces the risk of recurrence and breast cancer mortality [1]. The incidental radiation dose from the radiation treatment can, however, increase the risk of contralateral breast cancer [1–3]. Changes in radiotherapy delivery have successfully reduced this incidental dose [4], but given the widespread use of adjuvant radiotherapy, it is estimated that 17% of contralateral breast cancers could be related to incidental radiation exposure [4]. Hormone therapy is a highly effective treatment for ER+ breast cancer that reduces the risk of recurrence, breast cancer mortality, and contralateral breast cancer [5–7]. However, the combined effect of radiotherapy and hormone therapy on contralateral breast cancer risk is uncertain.

Most previous studies of radiotherapy and contralateral breast cancer included populations treated before the widespread introduction of hormone therapy [1, 2, 8, 9]. The Kaiser Permanente (KP) Breast Cancer Survivors Study is a cohort of breast cancer survivors diagnosed between 1990 and 2016 in a general community setting [10]. We used detailed treatment data from electronic medical records (EMRs) for hormone therapy, chemotherapy, and radiotherapy to estimate the combined effect of radiotherapy and hormone therapy on contralateral breast cancer. In the KP cohort, we estimated radiation dose to the contralateral breast and its association with contralateral breast cancer risk. This in-depth analysis was complemented with a large-scale population-based analysis in the SEER-9 cancer registries.

## Methods

### Study design and participants

#### KP cohort

**Fig. 1:**
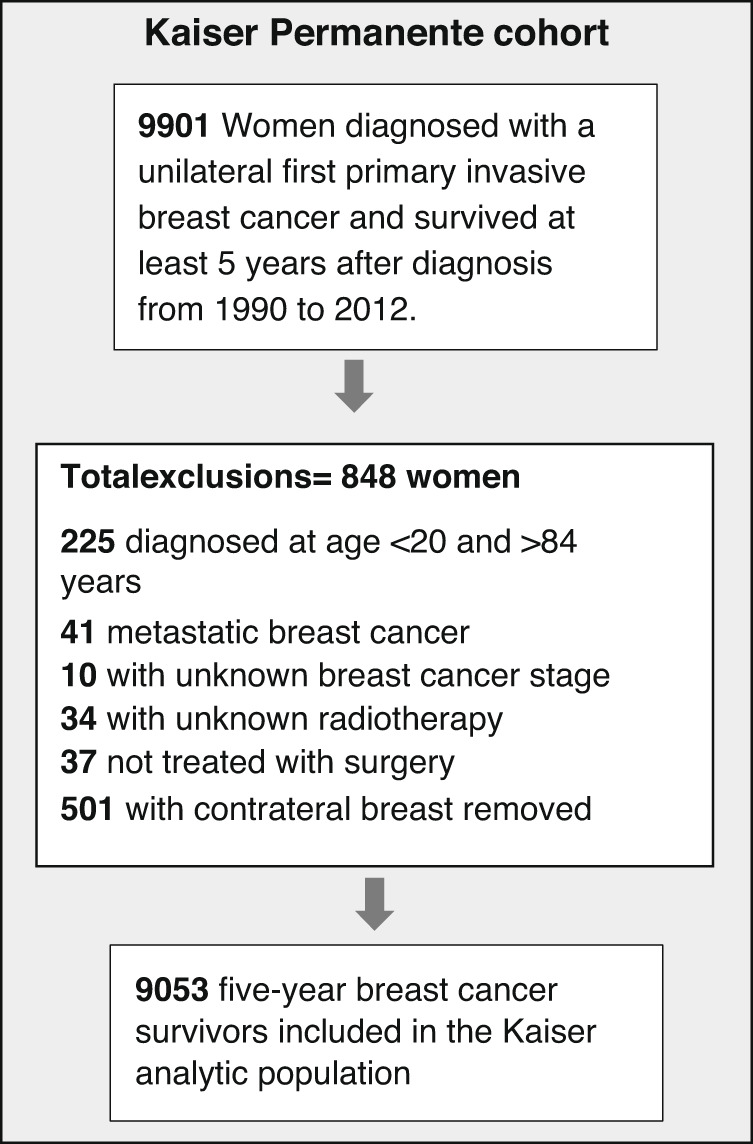
Diagram of selection for five-years breast cancer survivors in the Kaiser Permanente cohort.

The study population included 9901 breast cancer survivors diagnosed with unilateral first primary invasive breast cancer between 1990 and 2012 in three KP centres: KP Colorado (01/01/1994–12/31/2010), KP Northwest (01/01/1990–12/31/2005) and KP Washington (01/01/1990–12/31/2012). To account for the minimum latency period for radiation-related solid cancers [11], our study population was restricted to individuals who had survived for at least five years and cancer-free after diagnosis. Eligible subjects were women treated with surgery, with known stage (I-III), diagnosed age 20–84 years, and known radiotherapy and hormone therapy receipt. We excluded women who received prophylactic removal of the contralateral breast at initial surgery (Fig. 1). Our analytical population included 9053 five-year breast cancer survivors. Follow-up started five years after breast cancer diagnosis and continued until the earliest occurrence of an outcome event or one of the following censoring events: death, diagnosis of a second primary cancer diagnosis, KP disenrollment, or end of study (12/31/2015 for KP Colorado; 12/31/2017 for KP Washington; 12/31/2010 for KP Northwest).

The KP cohort study was approved by the Institutional Review Boards of each participating institution and the National Institutes of Health. A waiver of written informed consent was granted for the cohort creation based on the minimal risk of this electronic linkage-based research.

#### SEER cohort

We identified a population-based cohort of 289,394 women diagnosed with a unilateral first primary invasive breast cancer who survived and were cancer-free at least 5 years after diagnosis using the SEER-9 cancer registries from 1990–2013 [12]. We similarly restricted the SEER cohort to women treated with surgery, had a known stage (I-III), diagnosed at age 20–84 years, and excluded those who had removal of the contralateral breast at initial surgery. We also excluded patients for whom radiotherapy or hormone therapy was recommended, but unknown if administered, as well as patients with unknown information on hormone therapy, leaving 244,834 five-year breast cancer survivors (Supplementary Fig. 1). Follow-up started 5 years after breast cancer diagnosis until the earliest occurrence of an outcome event, second primary cancer diagnosis, death, last follow-up, or end of study (12/31/2018).

### Procedures

#### Data collection

For the KP cohort, we extracted data from EMRs, administrative billing and pharmacy dispensing records to ascertain patients’ demographic characteristics and initial breast cancer treatment (surgery type, radiotherapy, chemotherapy and hormone therapy). Patients were linked to local tumour registries or SEER Seattle-Puget-Sound (for KP Washington) to obtain tumour characteristics for the first primary breast cancer and all subsequent cancers. Vital status, date and cause of death were determined from the tumour registries and EMRs with additional linkage to the National Death Index for individuals with unknown cause of death (for KP Colorado).

Radiotherapy information in both the KP and SEER cohorts was ascertained from tumour registries and defined as any radiotherapy given as part of the initial breast cancer treatment. In SEER, hormone therapy was defined as any hormone therapy given as initial treatment or within one year of diagnosis. For KP, we included any hormone therapy prescription given from the date of diagnosis to the end of the study. Among ER+ patients who received hormone therapy, 94% started treatment within one year of diagnosis.

#### Radiation dosimetry in the KP cohort

We collected radiation summaries for all patients who received radiotherapy in the KP cohort. Digital copies were sent to the MD Anderson Late Effects Group, where a certified medical dosimetrist with expertise in late effects extracted treatment parameters for each radiotherapy field (e.g., machine, energy, treatment field types, wedge use and angles, delivered tumour dose and etc.). Individual contralateral breast dose reconstruction was performed for a subset of exposed subjects (*N* = 2442, 25%) as described elsewhere [13]. Briefly, contralateral breast doses were determined using a combination of radiation parameters and prior physical measurements [14] of absorbed dose in anthropomorphic phantoms. The average dose received by five subregions of the contralateral breast was estimated: nipple, upper inner quadrant (UIQ), lower inner quadrant (LIQ), upper outer quadrant (UOQ), and lower outer quadrant (LOQ).

For the current study, we estimated contralateral breast doses for all exposed subjects in the cohort by training a customised deep neural network using the previously reconstructed field-specific doses as ground truth and the corresponding abstracted radiotherapy parameters as input features. Based on prior research [15–18], our neural network consisted of 5 dense layers, each with a rectified linear unit activation function, and a dropout rate of 0.2. We used 60% of the data for training and 40% for held-out testing and a mean square dose error loss function. The model predicted dose to the five breast subregions from each radiotherapy field, enabling rapid dose estimation for the entire cohort without the need for manual, measurement-based reconstruction. On the held-out testing set, the coefficient of determination (R²) between the previously reconstructed doses and the predicted doses for each radiotherapy field was 0.98.

Total individual dose was obtained by summing contributions from all radiotherapy fields received by each patient. The mean dose to the whole contralateral breast was then calculated by averaging the doses to the five subregions. In 95% of patients with previously reconstructed doses, the predicted mean dose to the whole contralateral breast differed by less than 20 cGy. On average, the artificial intelligence method slightly overestimated the dose by 6 cGy (standard deviation 8 cGy) compared to the MD Anderson reconstruction, with a mean absolute difference of 8 cGy. Doses could not be estimated for 15% of the exposed patients (28 cases and 933 non-cases) due to either a missing radiation summary or incomplete information on radiation parameters.

#### Outcomes

The outcome of interest was a second primary cancer in the contralateral breast (including any invasive or in situ breast cancer) developed at least five years after the first breast cancer diagnosis until the end of follow-up.

### Statistical analysis

#### KP and SEER cohort studies

We estimated relative risk (RR) and 95% confidence Intervals (CIs) for contralateral breast cancer according to patient characteristics and radiotherapy and hormone therapy receipt using multivariable Poisson regression analysis. We used the expected number of breast cancers as an offset to indirectly adjust for attained age and attained calendar year [19]. The expected number of cases was estimated by multiplying the SEER-9 incidence rates of first breast cancer in the female population by the corresponding age, race, and period-specific person-years.

For the KP cohort, multivariable models were adjusted for age (20–39, 40–54, 55–69, 70–84) and year of diagnosis (1990–1997, 1998–2005, 2006–2012), radiotherapy (no/yes), chemotherapy (no/yes), and hormone therapy (no/yes) receipt and KP study centre (KP Colorado, KP Northwest and KP Washington). The SEER models were adjusted for age (20–39, 40–54, 55–69, 70–84) and diagnosis year (1990–1997, 1998–2005, 2006–2013), radiotherapy (no/unknown vs yes), chemotherapy (no/unknown vs yes), and hormone therapy (no/unknown vs yes). We additionally adjusted the SEER models for grade (1, 2, 3, 4, unknown) and tumour size (<1 cm, 1-<2 cm, 2-<5 cm, 5+ cm, and unknown) of the first breast cancer since these factors were also associated with contralateral breast cancer risk in SEER and could be an indication of misclassification of metastases as contralateral breast cancer in SEER. Adjustment factors were selected based on clinical importance or association with contralateral breast cancer risk in the multivariable analysis (*p* < 0.05).

In both cohorts, we evaluated the relationship of hormone therapy with radiotherapy restricted to patients who had an ER+ first breast cancer, i.e., eligible to receive hormone therapy. We estimated the risk of contralateral breast cancer after radiotherapy, stratified by whether patients received and did not receive hormone therapy, and reported the p-value for the heterogeneity of RR between the two subgroups (p-difference).

For the KP cohort, we also used multivariable Poisson regression to estimate relative risk and 95% CIs for contralateral breast cancer in relation to categories of mean radiation dose to the contralateral breast. Using dose as a continuous variable, we fit a regression model to evaluate whether there was a statistically significant linear association (p-trend <0.05) between the radiation dose and the occurrence of contralateral breast cancer. We also evaluated the radiation dose-response by hormone therapy receipt for each outcome and reported the p-value for heterogeneity of the ERR/Gy. Specifically, we used a linear model where the excess RR (ERR) = βD, in which the ERR is the RR minus 1, D is the equal dose, and β is the regression coefficient. Likelihood ratio-based tests and CIs were reported to assess the statistical significance and precision of our estimates.

P-values for heterogeneity of relative risks were estimated using nested likelihood ratio tests. All tests were 2-sided with statistical significance set at p < 0.05. Data were analysed using SEER*Stat 8.4.3, and DATAB, AMFIT and PECAN modules of Epicure (Hirosoft Corporation).

## Results

### KP cohort

There were 9053 five-year breast cancer survivors in the KP cohort. After a mean follow-up of 6 years (range: 0.1–22 years), 353 women developed contralateral breast cancer (259 ER+, 73%) (Table 1). Overall, about 70% of the cohort received radiotherapy, 66% had breast-conserving surgery, 38% received chemotherapy, and 83% of the women with ER+ breast cancer received hormone therapy.

**Table 1:** Selected patient and clinical characteristics among 9053 5-year breast cancer survivors in the Kaiser Permanente cohort.

Characteristics	Kaiser Permanente cohort									
	Non-cases (*N* = 8700)		All CBC (*N* = 353)		ER + CBC (*N* = 259)		ER- CBC (*N* = 58)		ER unknown CBC (*N* = 36)	
	*N*	%	*N*	%	*N*	%	*N*	%	*N*	%
**Age at 1st** **breast cancer diagnosis**										
20–39	252	2.9	20	5.7	13	5.0	5	8.6	2	5.6
40–54	2548	29.3	112	31.7	85	32.8	16	27.6	11	30.6
55–69	3641	41.9	159	45.0	113	43.6	32	55.2	14	38.9
70+	2259	26.0	62	17.6	48	18.5	5	8.6	9	25.0
**Attained age**^**a**^**, years**										
40–59	1524	17.5	76	21.5	50	19.3	15	25.9	11	30.6
60–69	2216	25.5	110	31.2	86	33.2	17	29.3	7	19.4
70–79	2353	27.0	102	28.9	74	28.6	17	29.3	11	30.6
80–97	2607	30.0	65	18.4	49	18.9	9	15.5	7	19.4
**Year of 1st** **breast cancer diagnosis**^**b**^										
1990–1993	1017	11.7	79	22.4	59	22.8	8	13.8	12	33.3
1994–1997	1760	20.2	98	27.8	68	26.3	15	25.9	15	41.7
1998–2001	2029	23.3	101	28.6	72	27.8	22	37.9	7	19.4
2002–2005	1942	22.3	46	13.0	37	14.3	8	13.8	1	2.8
2006–2012	1952	22.4	29	8.2	23	8.9	5	8.6	1	2.8
**Stage of the 1st** **breast cancer**										
I	5439	62.5	228	64.6	181	69.9	29	50.0	18	50.0
II	2774	31.9	106	30.0	68	26.3	22	37.9	16	44.4
III	487	5.6	19	5.4	10	3.9	7	12.1	2	5.6
**Grade of the 1st** **breast cancer**										
1	2498	28.7	89	25.2	74	28.6	8	13.8	7	19.4
2	3297	37.9	106	30.0	77	29.7	18	31.0	11	30.6
3	2037	23.4	90	25.5	55	21.2	26	44.8	9	25.0
4	55	0.6	4	1.1	3	1.2	0	0.0	1	2.8
Unknown	813	9.3	64	18.1	50	19.3	6	10.3	8	22.2
**Tumour size of 1st** **breast cancer, cm**										
<1	2191	25.2	96	27.2	79	30.5	8	13.8	9	25.0
1-<2	3786	43.5	149	42.2	112	43.2	25	43.1	12	33.3
2-<5	2357	27.1	93	26.3	59	22.8	20	34.5	14	38.9
5+	268	3.1	8	2.3	5	1.9	2	3.4	1	2.8
Unknown	98	1.1	7	2.0	4	1.5	3	5.2	0	0.0
**Histology of the 1st** **breast cancer**										
Ductal	6614	76.0	271	76.8	200	77.2	41	70.7	30	83.3
Lobular	733	8.4	29	8.2	17	6.6	8	13.8	4	11.1
Mixed	465	5.3	20	5.7	18	6.9	2	3.4	0	0.0
Others	788	9.1	33	9.3	24	9.3	7	12.1	2	5.6
**ER status of 1st** **breast cancer**										
ER+	6994	80.4	271	76.8	206	79.5	39	67.2	26	72.2
ER-	1306	15.0	58	16.4	33	12.7	17	29.3	8	22.2
ER unknown	400	4.6	24	6.8	20	7.7	2	3.4	2	5.6
**Surgery type**										
Breast conserving surgery	5750	66.1	237	67.1	179	69.1	38	65.5	20	55.6
Mastectomy	2950	33.9	116	32.9	80	30.9	20	34.5	16	44.4
**Radiotherapy**										
No	2626	30.2	105	29.7	78	30.1	11	19.0	16	44.4
Yes	6074	69.8	248	70.3	181	69.9	47	81.0	20	55.6
**Initial chemotherapy**										
No	5435	62.5	215	60.9	173	66.8	20	34.5	22	61.1
Yes	3265	37.5	138	39.1	86	33.2	38	65.5	14	38.9
**Hormone therapy**^**c**^										
No	2500	28.7	149	42.2	108	41.7	22	37.9	19	52.81
Yes	6500	71.3	204	57.8	151	58.3	36	62.1	17	47.2.4
**Hormone therapy (ER+ 1st** **breast cancer only)**^**d**^										
No	1204	17.2	83	30.6	66	32.0	7	17.9	10	38.5
Yes	5790	82.8	188	69.4	140	68.0	32	82.1	16	61.5

*CBC* contralateral breast cases, *ER* oestrogen receptor.

^a^Age at the exit date.

^b^The calendar period of 1994–2005 is the common period of breast cancer diagnosis for all Kaiser Permanente centres.

^c^Any hormone therapy received within the date of breast cancer diagnosis and exit date.

^d^Restricted to 6994 ER + first breast cancer patients.

Age at first breast cancer diagnosis was inversely associated with contralateral breast cancer risk (RR = 0.24, 95%CI: 0.14–0.41 for age 70+ vs age <40, p-trend<0.001). Overall, there was no relationship between ER-status, stage, grade, or tumour size of the first breast cancer and risk of contralateral breast cancer (Supplementary Table 1). Similar patterns were observed for ER+ and ER- contralateral breast cancer. Receipt of radiotherapy increased with year of diagnosis and stage at diagnosis, was more common after breast-conserving surgery than mastectomy, and was slightly more common with chemotherapy (Supplementary Table 2).

Overall, the relative risk of developing contralateral breast cancer was 1.07 (95%CI: 0.85–1.35) after radiotherapy; we observed a significantly decreased risk after hormone therapy (RR = 0.63, 95%CI:0.50–0.78) (Table 2). Radiotherapy receipt patterns were similar among those that did and did not receive hormone therapy (Supplementary Table 3). In those that did not receive hormone therapy, the risk of contralateral breast cancer after radiotherapy was 1.57 (95%CI:0.94–2.62), whereas there was no elevated risk of contralateral breast cancer after radiotherapy in those that also received hormone therapy (RR = 0.94, 95%CI: 0.69–1.29, p-difference = 0.09) (Table 2). For the risk of developing an ER+ contralateral breast cancer, these differences were even more marked and statistically significant; the RR for radiotherapy was 2.22 (95%CI:1.20–4.14) without hormone therapy and 0.88 (95%CI:0.61–1.26) with hormone therapy (p-difference = 0.01). The risk of ER- contralateral breast cancer was increased after radiotherapy (RR = 1.85, 95%CI:0.95–3.59).

**Table 2:** Relative risk (95%CI) for contralateral breast cancer according to treatment of first breast cancer among 5-year breast cancer survivors in the Kaiser cohort.

		Kaiser cohort			
Population	Treatment	Observed	RR^a^	95%CI	*P*-value^b^
		All Contralateral Breast Cases			
All first breast cancer	Radiotherapy				
	No	105	1		
	Yes	248	1.07	0.85–1.35	0.560
	Hormone therapy				
	No	149	1		
	Yes	204	0.63	0.50–0.78	<0.001
ER+ first breast cancer	Hormone therapy				
	No	83	1		
	Yes	188	0.53	0.40–0.70	<0.001
	Radiotherapy with HT				
	No	58	1		
	Yes	130	0.94^c^	0.69–1.29	0.717
	Radiotherapy without HT				
	No	21	1		
	Yes	62	1.57^c^	0.94–2.62	0.072
		ER+ Contralateral Breast Cases			
All first breast cancer patients	Radiotherapy				
	No	78	1		
	Yes	181	1.07	0.81–1.40	0.636
	Hormone therapy				
	No	108	1		
	Yes	151	0.63	0.48–0.81	<0.001
ER+ first breast cancer	Hormone therapy				
	No	66	1		
	Yes	140	0.52	0.38–0.71	<0001
	Radiotherapy with HT				
	No	46	1		
	Yes	94	0.88^d^	0.61–1.26	0.480
	Radiotherapy without HT				
	No	13	1		
	Yes	53	2.22^d^	1.20–4.14	0.007
		ER- Contralateral Breast Cases			
All first breast cancer	Radiotherapy				
	No	11	1		
	Yes	47	1.85	0.95–3.59	0.055
	Hormone therapy				
	No	22	1		
	Yes	36	0.72	0.42–1.23	0.235
ER+ first breast cancer	Hormone therapy				
	No	7	1		
	Yes	32	0.75	0.32–1.79	0.531

*RR* Relative Risk, *CI* confidence Interval, *ER* oestrogen receptor, *HT* hormone therapy.

^a^Relative risk estimated using Poisson regression, adjusted for year of diagnosis and age at diagnosis, radiotherapy, chemotherapy, hormone therapy, and study centre, as appropriate.

^b^Likelihood ratio test for heterogeneity of relative risks.

^c^P-difference = 0.09 between the RRs for radiotherapy with and without hormone therapy.

^d^P-difference = 0.01 between the RRs for radiotherapy with and without hormone therapy.

Among patients treated with radiotherapy, most were treated with tangential fields with a prescribed dose of 45-50 Gy delivered in fractions of 1.8-2 Gy to the breast or chest wall (Supplementary Table 4). Approximately 16% received additional fields, including supraclavicular and internal mammary node irradiation. Very few patients received hypo-fractionated radiotherapy (6%) or partial breast radiotherapy (<1%). The estimated dose to the contralateral breast was highest for the UIQ (mean = 1.55 Gy, range: 0.02–3.11) and lowest for the LOQ (mean = 0.60 Gy, range: 0.008–1.23). The mean average dose to the whole contralateral breast was 1.02 Gy (range: 0.015–2.08 Gy). Patients who received higher radiation doses are more likely to have been diagnosed at a younger age and higher stage, undergone mastectomy, and been treated in earlier years, and no difference with respect to hormone therapy receipt (Supplementary Table 5).

Overall, there was a statistically significant increase in ER- contralateral breast cancer risk with increasing radiation dose to the contralateral breast (ERR/Gy = 0.87, 95%CI: 0.04,2.72, p-trend = 0.033) (Table 3). A significant radiation dose-response (ERR/Gy = 1.37, 95% CI: 0.05, 5.85, p-trend = 0.036) was also observed among ER+ first breast cancer patients who received hormone therapy. However, the number of ER- cases among ER+ first breast cancer patients who did not receive hormone therapy was too small to yield meaningful results (*n* = 7). Among ER+ first breast cancer patients that received hormone therapy, the relative risk of developing an ER+ contralateral breast cancer associated with 1+ Gy versus 0 Gy was 0.97 (95%CI: 0.65–1.44) with no significant dose-response (ERR/Gy = -0.13, 95%CI:−0.36, 0.23, p-trend = 0.42). In contrast, among those who did not receive hormone therapy, the relative risk was 2.74 (95%CI:1.39–5.40) and significantly associated with radiation dose (ERR/Gy of 1.39 (95%CI:0.33–3.66, p-trend = 0.003). There was a significant heterogeneity of the ERR/Gy between ER+ first breast cancer patients who received and did not receive hormone therapy (p-difference = 0.006).

**Table 3:** - Relative risk (95%CI) for contralateral breast cancer according to treatment of first breast cancer among 5-year breast cancer survivors in the Kaiser cohort.

		Outcome											
Population	Contralateral breast dose, Gy (mean)	Observed	RR^a^	95%CI	P-trend^b^	Observed	RR^a^	95%CI	P-trend^b^	Observed	RR^a^	95%CI	P-trend^b^
		All Contralateral Breast Cases				ER+ Contralateral Breast Cases				ER- Contralateral Breast Cases			
**All first breast cancer**	**Radiotherapy (with and without HT)**												
	0	105	1			78	1			11	1		
	>0–0.99 (0.79)	65	0.94	0.68–1.30		49	0.97	0.67–1.41		10	1.27	0.52-3.09	
	1.0–2.11 (1.17)	155	1.15	0.90–1.49		112	1.14	0.85–1.53		30	2.04	1.02-4.09	
	Unknown dose	28	0.95	0.62–1.46		20	0.94	0.56–1.55		7	2.01	0.76-5.36	
	ERR/Gy (95%CI)	0.08 (−0.13, 0.34)			0.50	0.04 (−0.17, 0.35)			0.76	0.87 (0.04, 2.72)			0.033
**ER+ first breast cancer**	**Radiotherapy with HT**												
	0	58	1			46	1			5	1		
	>0–0.99 (0.79)	33	0.80	0.51–1.26		24	0.75	0.45–1.26		6	1.53	0.45-5.21	
	1.0–2.11 (1.17)	83	1.07	0.76–1.50		59	0.97	0.65–1.44		18	2.41	0.89-6.54	
	Unknown dose	14	0.72	0.39–1.31		11	0.76	0.39–1.51		3	1.31	0.31-5.63	
	ERR/Gy (95%CI)	−0.0004 (−0.24, 0.35)^c^			1.00	−0.13 (−0.36, 0.23)^d^			0.42	1.37 (0.05, 5.85)^e^			0.036
	**Radiotherapy without HT**												
	0	21	1			14	1			4	1		
	>0–0.99 (0.79)	18	1.52	0.78–2.98		11	1.97	0.90–4.31		1	0.64	0.06-6.83	
	1.0–2.11 (1.17)	38	1.86	1.06–3.27		36	2.74	1.39-–5.40		2	0.41	0.07-2.49	
	Unknown dose	6	1.22	0.47–3.13		5	2.04	0.74–5.63		0	-		
	ERR/Gy (95%CI)	0.67 (0.03, 1.84)^c^			0.038	1.39 (0.33, 3.66)^d^			0.0026	−0.58 (ne, 0.74)^e^			0.19

*RR* Relative Risk, *CI* confidence Interval, *ER* oestrogen receptor, *HT* hormone therapy, *ne* not estimable

^a^Relative risk estimated using Poisson regression, adjusted for year of diagnosis and age at diagnosis, radiotherapy, chemotherapy, hormone therapy, and study centre, as appropriate.

^b^Likelihood ratio test comparing a linear dose-response model with a baseline model (null model) using continuous dose.

^c^*P*-value = 0.16 for heterogeneity between the ERR/Gy for patients with and without hormone therapy receipt.

^d^*P*-value = 0.006 for heterogeneity between the ERR/Gy for patients with and without hormone therapy receipt.

^e^*P*-value = 0.12 for heterogeneity between the ERR/Gy for patients with and without hormone therapy receipt.

### SEER cohort

In the SEER cohort of 244,834 five-year breast cancer survivors, 10,470 developed a contralateral breast cancer (72% ER+) after a mean follow-up of 8 years (range 0.1–23 years) (Supplementary Table 6). A slightly lower proportion of patients underwent breast-conserving surgery (61%) than in the KP cohort (66%). Initial radiotherapy was 58% versus 70% in the KP cohort; a slightly lower proportion was classified as receiving initial chemotherapy (36% vs 38%), and a much lower proportion was classified as receiving hormone therapy (ER+ patients: 57% vs 83%).

The risk of contralateral breast cancer decreased with age and year of first breast cancer diagnosis (RR = 0.47, 95%CI:0.43–0.51 for 70+ vs <40 years, *p* < 0.001) (Supplementary Table 7). There was no association with stage of first breast cancer, but the risk was higher when the first breast cancer was ER- (compared to ER + ) (RR = 1.12, 95%CI:1.07–1.19). The risk for ER- contralateral breast cancer was increased after a first breast cancer of higher grade (*p* < 0.001) and larger tumour size (*p* < 0.029).

Overall, there was a small but statistically significantly increased risk of contralateral breast cancer after radiotherapy (yes vs no/unknown) (RR = 1.07, 95%CI: 1.03–1.12), and a decreased risk after hormone therapy (yes vs no/unknown) (RR = 0.92, 95%CI: 0.88–0.96) (Table 4). Among ER+ first breast cancer patients who did not receive hormone therapy, the risk of contralateral breast cancer after radiotherapy was 1.09 (95%CI:1.02–1.17) compared to 1.03 (95%CI:0.95–1.11) for those who received hormone therapy (p-difference=0.028). The risk of ER+ contralateral breast cancer after radiotherapy was 1.12 (95%CI:1.04–1.21) among those who did not receive hormone therapy vs 1.03 (95%CI:0.94–1.12) among those who did receive hormone therapy (p-difference = 0.16). For ER- contralateral breast cancer, the risk after radiotherapy was 1.12 (95%CI:1.01–1.23). The risk of contralateral breast cancer after radiotherapy was highest for the youngest women; among ER+ first breast cancer patients aged 20–39 years at diagnosis who did not receive hormone therapy the risk of ER+ contralateral breast cancer after radiotherapy was 1.37 (95%CI:0.97–1.95) and the overall risk of ER- contralateral breast cancer after radiotherapy was 1.31 (95% CI: 1.02–1.69) for the youngest women (Supplementary Table 8).

**Table 4:** Relative risk (95%CI) for contralateral breast cancer according to treatment of first breast cancer among 5-year breast cancer survivors in the SEER cohort.

		Outcome			
Population	Treatment	All Contralateral Breast Cases			
		Observed	RR^a^	95%CI	*P*-value^b^
All first breast cancer	Radiotherapy				
	No/Unknown^c^	4599	1		
	Yes	5871	1.07	1.03–1.12	<0.001
	Hormone therapy				
	No/Unknown^c^	6596	1		
	Yes	3874	0.92	0.88–0.96	<0.001
ER+ first breast cancer	Hormone therapy				
	No/Unknown^c^	3511	1		
	Yes	3421	0.95	0.90–0.99	0.039
	Radiotherapy with HT				
	No/Unknown^c^	1021	1		
	Yes	2400	1.03^d^	0.95–1.11	0.489
	Radiotherapy without HT				
	No/Unknown^c^	1784	1		
	Yes	1727	1.09^d^	1.02–1.17	0.010
		ER+ Contralateral Breast Cases			
All first breast cancer	Radiotherapy				
	No/Unknown^c^	3245	1		
	Yes	4345	1.07	1.02–1.12	0.006
	Hormone therapy				
	No/Unknown^c^	4618	1		
	Yes	2972	0.96	0.91–1.01	0.090
ER+ first breast cancer	Hormone therapy				
	No/Unknown^c^	2714	1		
	Yes	2679	0.94	0.89-0.99	0.035
	Radiotherapy with HT				
	No/Unknown^c^	785	1		
	Yes	1894	1.03^e^	0.94–1.12	0.414
	Radiotherapy without HT				
	No/Unknown^c^	1348			
	Yes	1366	1.12^e^	1.04–1.21	0.004
		ER- Contralateral Breast Cases			
All first breast cancer	Radiotherapy				
	No/Unknown^c^	799	1		
	Yes	1039	1.12	1.01–1.23	0.028
	Hormone therapy				
	No/Unknown^c^	1248	1		
	Yes	590	0.84	0.76–0.93	<0.001
ER+ first breast cancer	Hormone therapy				
	No/Unknown^c^	401	1		
	Yes	481	1.15	0.99–1.32	0.054
	Radiotherapy with HT				
	No/Unknown^c^	141	1		
	Yes	340	1.01 ^f^	0.83–1.24	0.69
	Radiotherapy without HT				
	No/Unknown^c^	203	1		
	Yes	198	1.10 ^f^	0.89–1.34	0.379

*BC* breast cancer, *ER* oestrogen receptor.

^a^Relative risk estimated using Poisson regression, adjusted for year of diagnosis and age at diagnosis, grade, tumour size, radiotherapy, chemotherapy, hormone therapy and grade and tumour size as appropriate.

^b^Likelihood ratio test for heterogeneity of relative risks.

^c^Treatment variables in SEER are classified as “yes” or “no/unknown” since SEER cannot accurately distinguish between “no treatment” and “unknown if ^p^atients received treatment”.

^d^P-difference=0.28 between the RRs for radiotherapy with and without hormone therapy.

^e^P-difference=0.16 between the RRs for radiotherapy with and without hormone therapy.

^f^P-difference=0.34 between the RRs for radiotherapy with and without hormone therapy.

Patterns of receipt of radiotherapy by patient and tumour characteristics and among those who did and did not receive hormone therapy were broadly similar to the KP cohort (Supplementary Tables 9 and 10).

## Discussion

We studied the risk of contralateral breast cancer after radiotherapy stratified by hormone therapy receipt in two US cohorts of breast cancer patients treated since the 1990s who survived at least 5 years. In the KP cohort, women with ER+ first breast cancer who did not receive hormone therapy had a significantly increased risk of ER+ contralateral breast cancer after radiotherapy (RR = 2.22), and the risk increased linearly with radiation dose to the contralateral breast. Nevertheless, there was no evidence of elevated risk associated with radiotherapy receipt and radiation dose among women who also received hormone therapy. The risk of ER- contralateral breast cancer was also increased after radiotherapy with a significant radiation dose-response. The patterns of risk estimates of radiotherapy stratified by hormone therapy were broadly consistent and statistically significant, although attenuated, in the larger general population analysis using the SEER cancer registries. The risk of radiation-related contralateral breast cancer was highest for women <age 40 at first breast cancer diagnosis.

Our study treatment period from 1990 to 2012 captured important changes, including the reduction of the incidental radiation dose to the contralateral breast [4], and the widespread introduction of hormone therapy. As far as we are aware, no other studies have reported findings for the risk of contralateral breast cancer associated with radiotherapy stratified by hormone therapy receipt. In the pooled analysis of randomised trials of radiotherapy and breast conserving-surgery the Early Breast Cancer Trialists’ Collaborative Group (EBCTCG) reported a significantly increased risk of contralateral breast cancer in women treated with radiotherapy (RR = 1.18, *p* = 0.002) with an excess cumulative risk of 1.8% by 15 years [5]. The risk after radiotherapy on those not treated with systemic therapy (chemotherapy or tamoxifen) was still increased (RR = 1.21, *p* = 0.01), but results were not presented for tamoxifen only. These trials were predominantly conducted in the 1970s and early 1980s using old radiotherapy regimens. In a large Dutch cohort of women treated between 1989 and 2002 [20], radiotherapy was not associated with an increased risk of contralateral breast cancer (RR = 1.04, 95%CI: 0.88–1.24). The authors noted that this could be because a large proportion of patients aged <45 years received hormone therapy (56%), but they did not present any results stratified by hormone therapy receipt. In the Dutch cohort analysis of women diagnosed from 2003–2010 [21], there was also no overall contralateral breast cancer risk associated with radiotherapy (RR = 0.94, 95%CI: 0.86–1.02). Still, results were not presented according to receipt of hormone therapy. A recent pooled analysis of BRCA mutation carriers found an increased risk of contralateral breast cancer after radiotherapy (RR = 1.44, 95%CI:1.12–1.86) [22]. The authors stated that there was no evidence of statistically significant interaction with hormone therapy, but also did not present risk estimates stratified by hormone therapy receipt, so we could not compare our findings. Additionally, a higher percentage of their study population was ER- (35%), and there was no analysis by contralateral breast cancer subtype.

There have been a few studies of radiotherapy and contralateral breast cancer that had detailed dosimetry to evaluate dose-response, but these studies were primarily conducted in earlier treatment periods and in younger women [2, 8, 9, 23, 24]. The WECARE case-control study [2] included women diagnosed between 1985-1999 and treated before age 55. The estimated mean dose was 1.1 Gy (range: 0.02-6.2 Gy) to the contralateral breast tumour location, and the OR for contralateral breast cancer was 2.5 for women diagnosed before age 40 who received >1 Gy compared to the non-exposed women. Our estimated radiation dose-response of an ERR/Gy of 0.87 for ER- contralateral breast cancer and 1.39 for ER+ contralateral breast cancer (among ER+ first breast cancer without hormone therapy) was similar in magnitude to their estimate of ERR/Gy of 1.0 (95%CI: 0.1–3.0). As the WECARE study focused on younger women, they included a larger proportion of ER- breast cancers (23%), and only 34% of their population received hormone therapy; they did not evaluate the radiation dose-response in relation to receipt of hormone therapy. In a Dutch cohort of women treated from 1970 to 1986, radiation doses were estimated for women treated before age 45. The authors reported a larger estimated dose for women treated with internal mammary chain fields [9]. We also found that the risk of radiation-related breast cancer was highest in the youngest women (age <40 at diagnosis) in the SEER cohort. Due to the smaller sample size in the KP cohort, we were unpowered to evaluate the age effect on the radiation risk. Other previous studies by Boice et al. (1992) [8], Storm et al. (1992) [24] and Basco et al. (1985) [23] included women of all ages diagnosed between 1935 and 1982 who were mostly treated with mastectomy rather than breast-conserving surgery and conducted prior to the widespread introduction of hormone therapy.

The role of endogenous oestrogens in the risk of radiation-related breast cancer is supported by the finding in other studies that radiation-related breast cancer risk is reduced in childhood cancer [25] and Hodgkin lymphoma survivors [26] who also received ovarian irradiation. A Phase-II clinical trial evaluated low-dose tamoxifen after chest irradiation in childhood cancer survivors and demonstrated reductions in breast density, which is a biomarker for breast cancer risk [27]. Further work on the potential for prevention of radiation-related ER+ breast cancer by hormone therapies is warranted.

Current US guidelines [28, 29] advise using hormone therapy for prevention in women at high risk of breast cancer. However, they do not extend this recommendation to women who have undergone chest irradiation, due to a lack of sufficient evidence. Our findings provide the first direct evidence that hormone therapy could be used to prevent radiation-related breast cancer.

The strengths of our two cohorts are complementary with detailed treatment data in the KP cohort and the large sample size and general population in SEER. Both also have long-term follow-up, which is required for studying radiation-related breast cancers that are known to take 5-10 years to develop. Assessment of the contralateral breast cancer by subtype enabled us to test the specificity of the risk after hormone therapy, and we adjusted for underlying trends in subtype-specific breast cancer rates using the general population rates as an offset in the Poisson regression model. Detailed treatment data for the KP cohort included radiotherapy summaries, which enabled us to evaluate the treatment techniques and estimate radiation doses to the contralateral breast. The results of the two studies were qualitatively similar, but the risk estimates were lower in SEER. It is well known that there is under-reporting of treatment in SEER, particularly for hormone therapy [30], which likely partly explains the lower risk estimates in SEER compared to the KP cohort, as non-differential misclassification usually biases risk estimates toward the null. It is also possible that there was misclassification of metastases as contralateral breast cancer [31], which could differ according to treatment and could bias results toward or away from the null. This could explain the association between contralateral breast cancer risk and tumour size and grade in SEER, which we included as adjustment factors. Also, as our data only includes the initial breast cancer treatment, we did not account for prophylactic mastectomy during follow-up. While some women may opt for this procedure later, this likely represents a small proportion, as prophylactic mastectomy at the time of initial surgery is often preferred for its single surgical event and recovery period. These limitations in conjunction with the smaller sample size in KP and wide confidence intervals, leave uncertainty about the magnitude of the risks. Overlap existed between the KP and SEER cohorts: 3692 (1.5%) patients from the KPWA cohort are included in the SEER cohort (*N* = 244,834). This overlap will therefore have a negligible impact on our results. We restricted our analysis to five-year survivors, resulting in the inclusion of only patients diagnosed up until 2013. This meant we had relatively few patients who received hypo-fractionated or partial-breast irradiation. The impact of these more recent changes in radiotherapy practice as well as the effect of the type of hormone therapy on radiation risk,warrants further investigation.

In summary, we observed an increased risk of ER- contralateral breast cancer after radiotherapy as well as an increased risk of ER+ contralateral breast cancer after radiotherapy in women who did not receive hormone therapy, with a significant radiation dose-response and higher radiation-related risk at younger ages. However, there was no associated risk of ER+ contralateral breast cancer from the incidental radiation dose in women who also received hormone therapy. This novel finding of potential prevention of radiation-related ER+ breast cancer by hormone therapy could have important clinical and public health implications, especially for individuals exposed to chest radiation for medical purposes (such as childhood cancer or Hodgkin lymphoma survivors).

## Supplementary information

Supplementary Data

## Data Availability

Data from this study, including individual participant data, are not available for sharing. Summary statistical data will be available from the corresponding author upon reasonable request, with the permission of the contributing Kaiser centres.
