# Supplementary material for: Contralateral breast cancer after radiotherapy and hormone therapy in two cohorts of US breast cancer survivors
Source: Br J Cancer. 2025 Nov 14;134(2):269–78. doi: 10.1038/s41416-025-03240-w (PMC12819377; doi:10.1038/s41416-025-03240-w)
Supplement: Supplementary file 1 — Supplementary Data [file 41416_2025_3240_MOESM1_ESM.docx]

**Supplementary Material**

**Contralateral breast cancer after radiotherapy and hormone therapy in two cohorts of US breast cancer survivors**

Lene HS Veiga, PhD, MS, Gretchen L Gierach, PhD, MPH, Susan A Smith, MPH, Rebecca M Howell, PhD, Matthew M Mille, PhD, Monjoy Saha, PhD, Rochelle E Curtis, MA, Cody Ramin, PhD, Clara Bodelon, PhD, Heather Spencer Feigelson, PhD, Erin J Aiello Bowles, MPH, Diana SM Buist, PhD, Sheila Weinmann, PhD, Jacqueline B Vo, PhD, RN, MPH, Choonsik Lee, PhD, Amy Berrington de Gonzalez, DPhil

**SEER cohort**

**289,394** Women diagnosed with a unilateral first primary invasive breast cancer who survived at least 5 years after diagnosis from 1990-2013.

**Total exclusions =44,560 women**

**7,130** diagnosed at age <20 and >84 years

**5,513** metastatic breast cancer

**2,818** unknown breast cancer stage

**4,508** with radiotherapy recommended, but unknown if administered.

**7,277** with hormone therapy recommended but unknown if administered or unknown if received.

**3,396** not treated with surgery.

**13,918** with contralateral breast removed.

**244,834** five-year breast cancer survivors included in the **SEER analytic population.**

Supplemental Figure 1 – Diagram of selection of five-year breast cancer survivors included in the SEER cohort.

| Supplemental Table 1 - Relative risk (95%CI) for contralateral breast cancer according to selected patient and clinical characteristics among 5-year breast cancer survivors in the Kaiser Permanente cohort. | | | | |
| --- | --- | --- | --- | --- |
|  | **Kaiser Permanente cohort** | | |  |
| **Characteristics** | **Observed** | **RR^a^** | **95%CI** | **P-value^b^** |
|  | **All Contralateral Breast Cases** | | | |
| **Overall** | 353 |  |  |  |
| **Age at 1^st^ breast cancer diagnosis** |  |  |  |  |
| 20-39 | 20 | 1 |  |  |
| 40-54 | 112 | 0.36 | 0.22-0.57 |  |
| 55-69 | 159 | 0.29 | 0.18-0.46 |  |
| 70-84 | 62 | 0.24 | 0.14-0.41 | <0.001 |
| **Year of 1^st^ breast cancer diagnosis** |  |  |  |  |
| 1990-1997 | 177 | 1 |  |  |
| 1998-2005 | 147 | 1.01 | 0.80-1.27 |  |
| 2006-2012 | 29 | 0.97 | 0.65-1.47 | 0.98 |
| **Stage of the 1^st^ breast cancer** |  |  |  |  |
| I | 228 | 1.00 |  |  |
| II | 106 | 1.09 | 0.83-1.44 |  |
| III | 19 | 1.18 | 0.70-1.96 | 0.76 |
| **Grade of the 1st breast cancer^c^** |  |  |  |  |
| 1 | 89 | 1 |  |  |
| 2 | 106 | 0.93 | 0.69-1.24 |  |
| 3 | 90 | 1.15 | 0.83-1.59 |  |
| 4 | 4 | 0.27 | 0.46-3.53 | 0.74 |
| **Tumor size of 1st breast cancer, cm^d^** |  |  |  |  |
| < 1 | 96 | 1 |  |  |
| 1-<2 | 149 | 0.99 | 0.76-1.30 |  |
| 2-<5 | 93 | 1.09 | 0.79-1.51 |  |
| 5+ | 8 | 0.72 | 0.34-1.53 | 0.67 |
| **Histology of the 1st breast cancer** |  |  |  |  |
| Ductal | 271 | 1 |  |  |
| Lobular | 29 | 1.18 | 0.79-1.75 |  |
| Mixed | 20 | 1.16 | 0.73-1.84 |  |
| Others | 33 | 0.85 | 0.59-1.24 | 0.74 |
| **ER status of 1st breast cancer ^e^** |  |  |  |  |
| ER+ | 271 | 1 |  |  |
| ER- | 58 | 1.08 | 0.80-1.45 |  |
| ER unknown | 24 | 1.23 | 0.80-1.88 | >0.50 |
|  | **ER+ Contralateral Breast Cases** | | | |
| **Overall** | 259 |  |  |  |
| **Age at 1^st^ breast cancer diagnosis** |  |  |  |  |
| 20-39 | 13 | 1 |  |  |
| 40-54 | 85 | 0.37 | 0.20-0.67 |  |
| 55-69 | 113 | 0.24 | 0.13-0.44 |  |
| 70+ | 48 | 0.22 | 0.11-0.41 | <0.001 |
| **Year of 1^st^ breast cancer diagnosis** |  |  |  |  |
| 1990-1997 | 127 | 1 |  |  |
| 1998-2005 | 109 | 0.98 | 0.75-1.28 |  |
| 2006-2012 | 23 | 1.01 | 0.64-1.61 | >0.50 |
| **Stage of the 1^st^ breast cancer** |  |  |  |  |
| I | 181 | 1.00 |  |  |
| II | 68 | 0.97 | 0.70-1.36 |  |
| III | 10 | 0.90 | 0.46-1.79 | 0.96 |
| **Grade of the 1st breast cancer^c^** |  |  |  |  |
| 1 | 74 | 1 |  |  |
| 2 | 77 | 0.83 | 0.60-1.16 |  |
| 3 | 55 | 0.88 | 0.59-1.32 |  |
| 4 | 3 | 1.21 | 0.37-3.95 | 0.69 |
| **Tumor size of 1st breast cancer, cm^d^** |  |  |  |  |
| < 1 | 79 | 1 |  |  |
| 1-<2 | 112 | 0.95 | 0.70-1.28 |  |
| 2-<5 | 59 | 0.94 | 0.64-1.38 |  |
| 5+ | 5 | 0.64 | 0.25-1.63 | 0.79 |
| **Histology of the 1st breast cancer** |  |  |  |  |
| Ductal | 198 | 1 |  |  |
| Lobular | 17 | 0.92 | 0.55-1.54 |  |
| Mixed | 18 | 1.39 | 0.85-2.28 |  |
| Others | 24 | 0.82 | 0.53-1.27 | 0.71 |
| **ER status of 1st breast cancer ^e^** |  |  |  |  |
| ER+ | 206 | 1 |  |  |
| ER- | 33 | 0.88 | 0.60-1.30 |  |
| ER unknown | 20 | 1.34 | 0.84-2.14 | 0.38 |
|  | **ER- Contralateral Breast Cases** | | | |
| **Overall** | 58 |  |  |  |
| **Age at 1^st^ breast cancer diagnosis** |  |  |  |  |
| 20-39 | 5 | 1 |  |  |
| 40-54 | 16 | 0.24 | 0.09-0.66 |  |
| 55-69 | 32 | 0.42 | 0.16-1.13 |  |
| 70+ | 5 | 0.22 | 0.06-0.80 | 0.030 |
| **Year of 1^st^ breast cancer diagnosis** |  |  |  |  |
| 1990-1997 | 23 | 1 |  |  |
| 1998-2005 | 30 | 1.20 | 0.68-2.12 |  |
| 2006-2012 | 5 | 0.98 | 0.36-2.68 | 0.79 |
| **Stage of the 1^st^ breast cancer** |  |  |  |  |
| I | 29 | 1.00 |  |  |
| II | 22 | 0.93 | 0.49-1.79 |  |
| III | 7 | 1.51 | 0.60-3.81 | 0.58 |
| **Grade of the 1st breast cancer^c^** |  |  |  |  |
| 1 | 8 | 1 |  |  |
| 2 | 18 | 1.57 | 0.67-3.66 |  |
| 3 | 26 | 2.67 | 1.10-6.44 |  |
| 4 | 0 | - |  | 0.066 |
| **Tumor size of 1st breast cancer, cm^d^** |  |  |  |  |
| < 1 | 8 | 1 |  |  |
| 1-<2 | 25 | 1.5 | 0.65-3.46 |  |
| 2-<5 | 20 | 1.61 | 0.65-3.96 |  |
| 5+ | 2 | 1.07 | 0.21-5.42 | 0.72 |
| **Histology of the 1st breast cancer** |  |  |  |  |
| Ductal | 39 | 1 |  |  |
| Lobular | 8 | 2.29 | 0.95-5.52 |  |
| Mixed | 2 | 0.75 | 0.16-3.49 |  |
| Others | 7 | 1.58 | 0.62-3.97 | 0.20 |
| **ER status of 1st breast cancer ^e^** |  |  |  |  |
| ER+ | 39 | 1 |  |  |
| ER- | 17 | 1.58 | 0.86-2.89 |  |
| ER unknown | 2 | 0.90 | 0.21-3.76 | 0.69 |
| Abbreviations: SIR, Standardized incidence ratio, ER,estrogen receptor; RT, Radiotherapy. | | | | |
| ^a^ Relative risk estimated using Poisson regression, adjusted for age and year of diagnosis, radiotherapy, chemotherapy, hormone therapy and study center, as appropriate.  ^b^ Likelihood ratio test for heterogeneity of relative risks. | | | | |
| ^c^ Excluded patients with unknown tumour grade (N=877)  ^d^ Excluded patients with unknown tumour size (N=105)  ^e^ Model did not include adjustment for hormone therapy | | | | |

| Supplemental Table 2 -Selected patient and clinical characteristics by radiotherapy status among non-cases in the Kaiser Permanente cohort. | | | | | | | | | | | |  |
| --- | --- | --- | --- | --- | --- | --- | --- | --- | --- | --- | --- | --- |
| **Characteristics of 1^st^ breast cancer** | **Kaiser cohort** | | | | | | | | | | | |
|  | **No RT** | | | | **RT** | | | | **Total** | | | |
|  | **N** | | **%** | | **N** | | **%** | | **N** | | **%** | |
| **Overall** | 2626 | | 29.5 | | 6074 | | 70.5 | | 8700 | | 100.0 | |
| **Age at diagnosis, years** |  | |  | |  | |  | |  | |  | |
| 20-39 | 75 | | 29.8 | | 177 | | 70.2 | | 252 | | 100.0 | |
| 40-54 | 662 | | 26.0 | | 1886 | | 74.0 | | 2548 | | 100.0 | |
| 55-69 | 1007 | | 27.7 | | 2634 | | 72.3 | | 3641 | | 100.0 | |
| 70-84 | 882 | | 39.0 | | 1377 | | 61.0 | | 2259 | | 100.0 | |
| **Age at diagnosis (ER+ only), years** | | | | | | | |  | |  | |  |
| 20-39 | 49 | | 30.8 | | 110 | | 69.2 | | 159 | | 100.0 | |
| 40-54 | 508 | | 25.7 | | 1468 | | 74.3 | | 1976 | | 100.0 | |
| 55-69 | 794 | | 26.6 | | 2190 | | 73.4 | | 2984 | | 100.0 | |
| 70-84 | 711 | | 37.9 | | 1164 | | 62.1 | | 1875 | | 100.0 | |
| **Year of diagnosis** |  | |  | |  | |  | |  | |  | |
| 1990-1993 | 407 | | 40.0 | | 610 | | 60.0 | | 1017 | | 100.0 | |
| 1994-1997 | 626 | | 35.6 | | 1134 | | 64.4 | | 1760 | | 100.0 | |
| 1998-2001 | 527 | | 26.0 | | 1502 | | 74.0 | | 2029 | | 100.0 | |
| 2002-2005 | 551 | | 28.4 | | 1391 | | 71.6 | | 1942 | | 100.0 | |
| 2006-2012 | 515 | | 26.4 | | 1437 | | 73.6 | | 1952 | | 100.0 | |
| **Stage** |  | |  | |  | |  | |  | |  | |
| I | 1575 | | 29.0 | | 3864 | | 71.0 | | 5439 | | 100.0 | |
| II | 958 | | 34.5 | | 1816 | | 65.5 | | 2774 | | 100.0 | |
| III | 93 | | 19.1 | | 394 | | 80.9 | | 487 | | 100.0 | |
| **Grade** | |  | |  | |  | |  | |  | |  |
| 1 | 638 | | 25.54 | | 1860 | | 74.5 | | 2498 | | 100.0 | |
| 2 | 1027 | | 31.15 | | 2270 | | 68.9 | | 3297 | | 100.0 | |
| 3 | 587 | | 28.82 | | 1450 | | 71.2 | | 2037 | | 100.0 | |
| 4 | 17 | | 30.91 | | 38 | | 69.1 | | 55 | | 100.0 | |
| Unknown | 357 | | 43.91 | | 456 | | 56.1 | | 813 | | 100.0 | |
| **Tumor size, cm** | | | |  | |  | |  | |  | |  |
| < 1 | 614 | | 28.02 | | 1577 | | 72.0 | | 2191 | | 100.0 | |
| 1-<2 | 1037 | | 27.39 | | 2749 | | 72.6 | | 3786 | | 100.0 | |
| 2-<5 | 865 | | 36.7 | | 1492 | | 63.3 | | 2357 | | 100.0 | |
| 5+ | 71 | | 26.39 | | 198 | | 73.6 | | 269 | | 100.0 | |
| Unknown | 40 | | 40.82 | | 58 | | 59.2 | | 98 | | 100.0 | |
| **ER status** | |  | |  | |  | |  | |  | |  |
| ER+ | 2062 | | 29.5 | | 4932 | | 70.5 | | 6994 | | 100.0 | |
| ER- | 410 | | 31.4 | | 896 | | 68.6 | | 1306 | | 100.0 | |
| ER unknown | 154 | | 38.5 | | 246 | | 61.5 | | 400 | | 100.0 | |
| **Surgery type** |  | |  | |  | |  | |  | |  | |
| Breast conserving surgery | 349 | | 6.1 | | 5401 | | 93.9 | | 5750 | | 100.0 | |
| Mastectomy | 2277 | | 77.2 | | 673 | | 22.8 | | 2950 | | 100.0 | |
| **Chemotherapy** |  | |  | |  | |  | |  | |  | |
| No | 1768 | | 32.5 | | 3667 | | 67.5 | | 5435 | | 100.0 | |
| Yes | 858 | | 26.3 | | 2407 | | 73.7 | | 3265 | | 100.0 | |
| Abbreviations: ER-Estrogen receptor; RT- Radiotherapy | | | | | | | | | | | |  |

| Supplemental Table 3 - Selected patient and clinical characteristics of 5-year breast cancer survivors by radiotherapy status according to hormone therapy receipt in the Kaiser Permanente cohort. Restricted to ER+ non-case patients. | | | | | | | | | | | | | | | | | | | |
| --- | --- | --- | --- | --- | --- | --- | --- | --- | --- | --- | --- | --- | --- | --- | --- | --- | --- | --- | --- |
|  | | **Kaiser cohort** | | | | | | | | | | | | | | | | | |
|  | | **No Hormone therapy** | | | | | | | |  | **Hormone therapy** | | | | | | | | |
|  | | **No RT** | | **RT** | | **Total** | | | |  | **No RT** | | | **RT** | | | **Total** | | |
| **Characteristics of 1^st^ breast cancer patients** | | **N** | **%** | **N** | **%** | **N** | | **%** | |  | **N** | **%** | | **N** | | **%** | **N** | | **%** |
| Overall | | 389 | 32.3 | 815 | 67.7 | 1204 | | 100.0 | |  | 1673 | 28.9 | | 4117 | | 71.1 | 5790 | | 100.0 |
| **Age at diagnosis** | |  |  |  |  |  | |  | |  |  |  | |  | |  |  | |  |
| 20-39 | | 7 | 28.0 | 18 | 72.0 | 25 | | 100.0 | |  | 42 | 31.3 | | 92 | | 68.7 | 134 | | 100.0 |
| 40-54 | | 78 | 30.2 | 180 | 69.8 | 258 | | 100.0 | |  | 430 | 25.0 | | 1,288 | | 75.0 | 1718 | | 100.0 |
| 55-69 | | 105 | 24.9 | 316 | 75.1 | 421 | | 100.0 | |  | 689 | 26.9 | | 1,874 | | 73.1 | 2563 | | 100.0 |
| 70-84 | | 199 | 39.8 | 301 | 60.2 | 500 | | 100.0 | |  | 512 | 37.2 | | 863 | | 62.8 | 1375 | | 100.0 |
| **Year of diagnosis** | |  |  |  |  |  | |  | |  |  |  | |  | |  |  | |  |
| 1990-1993 | | 68 | 38.0 | 111 | 62.0 | 179 | | 100.0 | |  | 229 | 41.0 | | 330 | | 59.0 | 559 | | 100.0 |
| 1994-1997 | | 88 | 28.5 | 221 | 71.5 | 309 | | 100.0 | |  | 395 | 37.0 | | 672 | | 63.0 | 1067 | | 100.0 |
| 1998-2001 | | 86 | 32.6 | 178 | 67.4 | 264 | | 100.0 | |  | 327 | 23.9 | | 1,042 | | 76.1 | 1,369 | | 100.0 |
| 2002-2005 | | 81 | 32.0 | 172 | 68.0 | 253 | | 100.0 | |  | 362 | 26.6 | | 997 | | 73.4 | 1359 | | 100.0 |
| 2006-2012 | | 66 | 33.2 | 133 | 66.8 | 199 | | 100.0 | |  | 360 | 25.1 | | 1,076 | | 74.9 | 1436 | | 100.0 |
| **Stage** | |  |  |  |  |  | |  | |  |  |  | |  | |  |  | |  |
| I | | 322 | 30.5 | 734 | 69.5 | 1056 | | 100.0 | |  | 915 | 26.7 | | 2,509 | | 73.3 | 3424 | | 100.0 |
| II | | 61 | 47.7 | 67 | 52.3 | 128 | | 100.0 | |  | 690 | 34.2 | | 1,325 | | 65.8 | 2015 | | 100.0 |
| III | | 6 | 30.0 | 14 | 70.0 | 20 | | 100.0 | |  | 68 | 19.4 | | 283 | | 80.6 | 351 | | 100.0 |
| **Grade** | |  |  |  |  |  | |  | |  |  |  | |  | |  |  | |  |
| 1 | | 141 | 26.6 | 389 | 73.4 | 530 | | 100.0 | |  | 442 | 24.4 | | 1371 | | 75.6 | 1813 | | 100.0 |
| 2 | | 152 | 35.1 | 281 | 64.9 | 433 | | 100.0 | |  | 752 | 29.9 | | 1762 | | 70.1 | 2514 | | 100.0 |
| 3 | | 45 | 38.5 | 72 | 61.5 | 117 | | 100.0 | |  | 272 | 27.4 | | 721 | | 72.6 | 993 | | 100.0 |
| 4 | | 1 | 50.0 | 1 | 50.0 | 2 | | 100.0 | |  | 8 | 32.0 | | 17 | | 68.0 | 25 | | 100.0 |
| Unknown | | 50 | 41.0 | 72 | 59.0 | 122 | | 100.0 | |  | 199 | 44.7 | | 246 | | 55.3 | 445 | | 100.0 |
| **Tumor size, cm** | | |  |  |  |  | |  | |  |  |  | |  | |  |  | |  |
| < 1 | | 164 | 26.9 | 445 | 73.1 | 609 | | 100.0 | |  | 292 | 25.4 | | 856 | | 74.6 | 1148 | | 100.0 |
| 1-<2 | | 155 | 34.1 | 300 | 65.9 | 455 | | 100.0 | |  | 710 | 25.8 | | 2045 | | 74.2 | 2755 | | 100.0 |
| 2-<5 | | 59 | 48.0 | 64 | 52.0 | 123 | | 100.0 | |  | 609 | 36.9 | | 1040 | | 63.1 | 1649 | | 100.0 |
| 5+ | | 6 | 60.0 | 4 | 40.0 | 10 | | 100.0 | |  | 42 | 22.7 | | 143 | | 77.3 | 185 | | 100.0 |
| Unknown | | 5 | 71.4 | 2 | 28.6 | 7 | | 100.0 | |  | 20 | 37.7 | | 33 | | 62.3 | 53 | | 100.0 |
| **Surgery type** | |  |  |  |  |  | |  | |  |  |  | |  | |  |  | |  |
| Breast-conserving surgery | | 106 | 11.7 | 797 | 88.3 | 903 | | 100.0 | |  | 179 | 4.7 | | 3,645 | | 95.3 | 3824 | | 100.0 |
| Mastectomy | | 283 | 94.0 | 18 | 6.0 | 301 | | 100.0 | |  | 1,494 | 76.0 | | 472 | | 24.0 | 1966 | | 100.0 |
| **Chemotherapy** | |  |  |  |  |  | |  | |  |  |  | |  | |  |  | |  |
| No | | 348 | 32.9 | 711 | 67.1 | 1059 | | 100.0 | |  | 1,123 | 30.5 | | 2,555 | | 69.5 | 3678 | | 100.0 |
| Yes | | 41 | 28.3 | 104 | 71.7 | 145 | | 100.0 | |  | 550 | 26.0 | | 1,562 | | 74.0 | 2112 | | 100.0 |
| Abbreviations: ER, estrogen receptor; RT, radiotherapy | | | | | | | | | | | | | | | | | | | |
| Supplemental table 4 - Radiotherapy parameters and estimated contralateral breast doses among KP 5-years survivors cohort^a^. | | | | | | | | | | | | | | | | |  |  |  |
|  | | | | | | Overall cohort | | | | | | All CBC | | | | |  |  |  |
| Radiotherapy fields | | | | | |  | |  | | | |  | |  | | |  |  |  |
| Breast/chestwall | | | | | | 4402 | | 80.6 | | | | 186 | | 83.4 | | |  |  |  |
| Other fields | | | | | | 1016 | | 18.6 | | | | 36 | | 16.1 | | |  |  |  |
| Unknown | | | | | | 43 | | 0.8 | | | | 1 | | 0.4 | | |  |  |  |
| Daily fractionated dose*, Gy | | | | | |  | |  | | | |  | |  | | |  |  |  |
| 1.8 | | | | | | 3109 | | 56.9 | | | | 131 | | 58.7 | | |  |  |  |
| 2.0 | | | | | | 1353 | | 24.8 | | | | 52 | | 23.3 | | |  |  |  |
| ≥2.6 to ≤3.8 | | | | | | 399 | | 7.3 | | | | 5 | | 2.2 | | |  |  |  |
| Others | | | | | | 41 | | 0.8 | | | | 3 | | 1.3 | | |  |  |  |
| Unknown | | | | | | 559 | | 10.2 | | | | 32 | | 14.3 | | |  |  |  |
| Partial breast irradiation | | | | | |  | |  | | | |  | |  | | |  |  |  |
| No | | | | | | 5391 | | 98.7 | | | | 222 | | 99.6 | | |  |  |  |
| Yes | | | | | | 21 | | 0.4 | | | | 0 | | 0.0 | | |  |  |  |
| Unknowm | | | | | | 49 | | 0.9 | | | | 1 | | 0.4 | | |  |  |  |
| Prescribed dose, Gy | | | | | |  | |  | | | |  | |  | | |  |  |  |
| 42.6 | | | | | | 371 | | 6.8 | | | | 5 | | 2.2 | | |  |  |  |
| 45 | | | | | | 339 | | 6.2 | | | | 11 | | 4.9 | | |  |  |  |
| 46 | | | | | | 282 | | 5.2 | | | | 3 | | 1.3 | | |  |  |  |
| 50.0 or 50.4 | | | | | | 4017 | | 73.6 | | | | 187 | | 83.9 | | |  |  |  |
| Unknown | | | | | | 452 | | 8.3 | | | | 17 | | 7.6 | | |  |  |  |
| Contralateral breast sub-region dose, Gy ^b^ | | | | | | Mean (SD) | | Range | | | | Mean (SD) | | Range | | |  |  |  |
| Upper inner quadrant | | | | | | 1.55 (0.36) | | 0.02-3.11 | | | | 1.61 (0.33) | | 0.03-2.37 | | |  |  |  |
| Lower inner quadrant | | | | | | 1.38 (0.32) | | 0.03-2.80 | | | | 1.44 (0.30) | | 0.03-2.16 | | |  |  |  |
| nipple or central | | | | | | 1.03 (0.26) | | 0.01-2.13 | | | | 1.08 (0.24) | | 0.015-1.60 | | |  |  |  |
| Upper outer quadrant | | | | | | 0.64 (0.16) | | 0.007-1.31 | | | | 0.67 (0.15) | | 0.008-1.01 | | |  |  |  |
| Lower outer quadrant | | | | | | 0.60 (0.15) | | 0.008-1.23 | | | | 0.63 (0.14) | | 0.009-0.94 | | |  |  |  |
| Whole breast dose, Gy | | | | | | 1.02 (0.24) | | 0.015-2.08 | | | | 1.05 (0.22) | | (0.02-1.49) | | |  |  |  |
| ^a^ Includes patients who received radiotherapy and with a radiation summary available (5461 out of 6,322) | | | | | | | | | | | | | | | | |  |  |  |
| ^b^ Among exposed patients with known radiation dose (5361 out of 5461) | | | | | | | | | | | |  | |  | | |  |  |  |

| Supplemental Table 5 - Distribution of selected patient and tumor characteristics by contralateral breast dose among exposed patients with known radiation dose in the KP cohort | | | | | | |
| --- | --- | --- | --- | --- | --- | --- |
|  | **Radiation dose categories, Gy** | | | | | |
| **Characteristics of 1^st^ breast cancer patients** | **>0 - <1.0** | | **≥1.0** | | **Overall** | |
|  | **N** | **%** | **N** | **%** | **N** | **%** |
| **Overall** | 2191 | 40.9 | 3170 | 59.1 | 5361 | 100.0 |
| **Age at diagnosis, years** |  |  |  |  |  |  |
| <50 | 406 | 36.6 | 704 | 63.4 | 1110 | 100.0 |
| 50-59 | 622 | 40.7 | 908 | 59.3 | 1530 | 100.0 |
| 60+ | 1163 | 42.7 | 1558 | 57.3 | 2721 | 100.0 |
| **Year of diagnosis** |  |  |  |  |  |  |
| 1990-1997 | 396 | 23.9 | 1259 | 76.1 | 1655 | 100.0 |
| 1998-2005 | 1054 | 41.2 | 1506 | 58.8 | 2560 | 100.0 |
| 2006-2012 | 741 | 64.7 | 405 | 35.3 | 1146 | 100.0 |
| **Stage** |  |  |  |  |  |  |
| I | 1501 | 43.7 | 1933 | 56.3 | 3434 | 100.0 |
| II | 565 | 35.7 | 1019 | 64.3 | 1584 | 100.0 |
| III | 125 | 36.4 | 218 | 63.6 | 343 | 100.0 |
| Unknown |  |  |  |  |  |  |
| **Grade** |  |  |  |  |  |  |
| 1 | 744 | 45.1 | 906 | 54.9 | 1650 | 100.0 |
| 2 | 836 | 42.7 | 1124 | 57.3 | 1960 | 100.0 |
| 3 | 495 | 39.0 | 773 | 61.0 | 1268 | 100.0 |
| 4 | 16 | 45.7 | 19 | 54.3 | 35 | 100.0 |
| Unknown | 100 | 22.3 | 348 | 77.7 | 448 | 100.0 |
| **Estrogen receptor (ER) status** | | |  |  |  |  |
| ER- | 318 | 39.3 | 492 | 60.7 | 810 | 100.0 |
| ER+ | 1804 | 41.7 | 2521 | 58.3 | 4325 | 100.0 |
| Unknown | 69 | 30.5 | 157 | 69.5 | 226 | 100.0 |
| **Chemotherapy** |  |  |  |  |  |  |
| No | 1389 | 42.9 | 1852 | 57.1 | 3241 | 100.0 |
| Yes | 802 | 37.8 | 1318 | 62.2 | 2120 | 100.0 |
| **Any hormone therapy (ER+ only)** |  |  |  |  |  |  |
| No | 319 | 43.2 | 419 | 56.8 | 738 | 100.0 |
| Yes | 1485 | 41.4 | 2102 | 58.6 | 3587 | 100.0 |
| **Surgery type** |  |  |  |  |  |  |
| Breast conserving surgery | 1994 | 41.7 | 2792 | 58.3 | 4786 | 100.0 |
| Mastectomy | 197 | 34.3 | 378 | 65.7 | 575 | 100.0 |

| Supplemental Table 6 - Selected patient and clinical characteristics among 5-year breast cancer survivors in the SEER cohort | | | | | | | | | | | | | | | | | | |  |
| --- | --- | --- | --- | --- | --- | --- | --- | --- | --- | --- | --- | --- | --- | --- | --- | --- | --- | --- | --- |
| **Characteristics of 1^st^ breast cancer patients** | **SEER cohort** | | | | | | | | | | | | | | | | | | |
|  | **Non-cases (N=234,364)** | | | **All CBC (N=10,470)** | | | | **ER+ CBC (N=7590)** | | | | **ER- CBC (N=1838)** | | | | **ER unknown CBC (N=1042)** | | | |
|  | **N** | **%** | | **N** | | **%** | | **N** | | **%** | | **N** | | **%** | | **N** | | **%** | |
| **Age at diagnosis, years** |  |  | |  | |  | |  | |  | |  | |  | |  | |  | |
| 20-39 | 13,127 | 5.6 | | 817 | | 7.8 | | 428 | | 5.6 | | 288 | | 15.7 | | 101 | | 9.7 | |
| 40-54 | 76,533 | 32.7 | | 3,876 | | 37.0 | | 2750 | | 36.2 | | 756 | | 41.1 | | 370 | | 35.5 | |
| 55-69 | 88,210 | 37.6 | | 4,208 | | 40.2 | | 3228 | | 42.5 | | 595 | | 32.4 | | 385 | | 36.9 | |
| 70-84 | 56,494 | 24.1 | | 1,569 | | 15.0 | | 1184 | | 15.6 | | 199 | | 10.8 | | 186 | | 17.9 | |
| **Attained age, years** |  |  | |  | |  | |  | |  | |  | |  | |  | |  | |
| 40-59 | 66,678 | 28.5 | | 2,587 | | 24.7 | | 1552 | | 20.4 | | 693 | | 37.7 | | 342 | | 32.8 | |
| 60-69 | 65,382 | 27.9 | | 3,051 | | 29.1 | | 2264 | | 29.8 | | 535 | | 29.1 | | 252 | | 24.2 | |
| 70-79 | 60,776 | 25.9 | | 2,966 | | 28.3 | | 2314 | | 30.5 | | 387 | | 21.1 | | 265 | | 25.4 | |
| 80-105 | 41,528 | 17.7 | | 1,866 | | 17.8 | | 1460 | | 19.2 | | 223 | | 12.1 | | 183 | | 17.6 | |
| **Year of diagnosis** |  |  | |  | |  | |  | |  | |  | |  | |  | |  | |
| 1990-1993 | 33,546 | 14.3 | | 2,419 | | 23.1 | | 1,605 | | 21.1 | | 389 | | 21.2 | | 425 | | 40.8 | |
| 1994-1997 | 37,208 | 15.9 | | 2,585 | | 24.7 | | 1,814 | | 23.9 | | 468 | | 25.5 | | 303 | | 29.1 | |
| 1998-2001 | 41,988 | 17.9 | | 2,449 | | 23.4 | | 1,827 | | 24.1 | | 447 | | 24.3 | | 175 | | 16.8 | |
| 2002-2005 | 39,975 | 17.1 | | 1,694 | | 16.2 | | 1,305 | | 17.2 | | 304 | | 16.5 | | 85 | | 8.2 | |
| 2006-2009 | 40,587 | 17.3 | | 1,010 | | 9.6 | | 796 | | 10.5 | | 170 | | 9.2 | | 44 | | 4.2 | |
| 2010-2013 | 41,060 | 17.5 | | 313 | | 3.0 | | 243 | | 3.2 | | 60 | | 3.3 | | 10 | | 1.0 | |
| **Stage** |  |  | |  | |  | |  | |  | |  | |  | |  | |  | |
| Localised | 168,517 | 71.9 | | 7,632 | | 72.9 | | 5,565 | | 73.3 | | 1297 | | 70.6 | | 770 | | 73.9 | |
| Regional | 65,847 | 28.1 | | 2,838 | | 27.1 | | 2,025 | | 26.7 | | 541 | | 29.4 | | 272 | | 26.1 | |
| **Grade** |  |  | |  | |  | |  | |  | |  | |  | |  | |  | |
| 1 | 47,514 | 20.3 | | 1,893 | | 18.1 | | 1,526 | | 20.1 | | 203 | | 11.0 | | 164 | | 15.7 | |
| 2 | 90,355 | 38.6 | | 3,567 | | 34.1 | | 2,746 | | 36.2 | | 478 | | 26.0 | | 343 | | 32.9 | |
| 3 | 65,845 | 28.1 | | 3,133 | | 29.9 | | 2,018 | | 26.6 | | 839 | | 45.6 | | 276 | | 26.5 | |
| 4 | 3,358 | 1.4 | | 228 | | 2.2 | | 143 | | 1.9 | | 55 | | 3.0 | | 30 | | 2.9 | |
| Unknown | 27,292 | 11.6 | | 1,649 | | 15.7 | | 1,157 | | 15.2 | | 263 | | 14.3 | | 229 | | 22.0 | |
| **Tumor size, cm** |  |  | |  | |  | |  | |  | |  | |  | |  | |  | |
| < 1 | 53,862 | 23.0 | | 2,375 | | 22.7 | | 1,815 | | 23.9 | | 329 | | 17.9 | | 231 | | 22.2 | |
| 1-<2 | 91,429 | 39.0 | | 4,056 | | 38.7 | | 3,022 | | 39.8 | | 642 | | 34.9 | | 392 | | 37.6 | |
| 2-<5 | 70,440 | 30.1 | | 3,104 | | 29.6 | | 2,130 | | 28.1 | | 675 | | 36.7 | | 299 | | 28.7 | |
| 5+ | 10,881 | 4.6 | | 504 | | 4.8 | | 336 | | 4.4 | | 112 | | 6.1 | | 56 | | 5.4 | |
| Unknown | 7,752 | 3.3 | | 431 | | 4.1 | | 287 | | 3.8 | | 80 | | 4.4 | | 64 | | 6.1 | |
| **Histology** |  |  | |  | |  | |  | |  | |  | |  | |  | |  | |
| Ductal | 185,420 | 79.1 | | 7,694 | | 73.5 | | 5,528 | | 72.8 | | 1,417 | | 77.1 | | 749 | | 71.9 | |
| Lobular | 19,939 | 8.5 | | 800 | | 7.6 | | 629 | | 8.3 | | 101 | | 5.5 | | 70 | | 6.7 | |
| Mixed/others | 15,250 | 6.5 | | 779 | | 7.4 | | 602 | | 7.9 | | 88 | | 4.8 | | 89 | | 8.5 | |
| Others | 24,225 | 10.3 | | 1,197 | | 11.4 | | 831 | | 10.9 | | 232 | | 12.6 | | 134 | | 12.9 | |
| **ER status of first breast cancer** |  |  | |  | |  | |  | |  | |  | |  | |  | |  | |
| ER+ | 171,592 | 73.2 | | 6,932 | | 66.2 | | 5393 | | 71.1 | | 882 | | 48.0 | | 657 | | 63.1 | |
| ER- | 39,830 | 17.0 | | 2,215 | | 21.2 | | 1274 | | 16.8 | | 729 | | 39.7 | | 212 | | 20.3 | |
| ER unknown | 22,942 | 9.8 | | 1,323 | | 12.6 | | 923 | | 12.2 | | 227 | | 12.4 | | 173 | | 16.6 | |
| **Surgery type** |  |  | |  | |  | |  | |  | |  | |  | |  | |  | |
| BCS | 142,299 | 60.7 | | 6,020 | | 57.5 | | 4458 | | 58.7 | | 1039 | | 56.5 | | 523 | | 50.2 | |
| Mastectomy | 91,961 | 39.2 | | 4,447 | | 42.5 | | 3130 | | 41.2 | | 799 | | 43.5 | | 518 | | 49.7 | |
| Surgery, NOS | 104 | 0.0 | | 3 | | 0.0 | | 2 | | 0.0 | | 0 | | 0.0 | | 1 | | 0.1 | |
| **Radiotherapy** |  |  | |  | |  | |  | |  | |  | |  | |  | |  | |
| No/Unknown | 98,230 | 41.9 | | 4,599 | | 43.9 | | 3245 | | 42.8 | | 799 | | 43.5 | | 555 | | 53.3 | |
| Yes | 136,134 | 58.1 | | 5,871 | | 56.1 | | 4345 | | 57.2 | | 1039 | | 56.5 | | 487 | | 46.7 | |
| **Chemotherapy** |  |  | |  | |  | |  | |  | |  | |  | |  | |  | |
| No/Unknown | 149,285 | 63.7 | | 6,533 | | 62.4 | | 4899 | | 64.5 | | 915 | | 49.8 | | 719 | | 69.0 | |
| Yes | 85,079 | 36.3 | | 3,937 | | 37.6 | | 2691 | | 35.5 | | 923 | | 50.2 | | 323 | | 31.0 | |
| **Hormone therapy** |  |  | |  | |  | |  | |  | |  | |  | |  | |  | |
| No/Unknown | 128,239 | 74.7 | | 6,596 | | 95.2 | | 4618 | | 85.6 | | 1248 | | 141.5 | | 730 | | 111.1 | |
| Yes | 106,125 | 61.8 | | 3,874 | | 55.9 | | 2972 | | 55.1 | | 590 | | 66.9 | | 312 | | 47.5 | |
| **Hormone therapy (ER+ only)^a^** | | | 10,470 | |  | |  | |  | |  | |  | |  | |  | |  |
| No/Unknown | 73,788 | 43.0 | | 3,511 | | 50.6 | | 2714 | | 50.3 | | 401 | | 45.5 | | 396 | | 60.3 | |
| Yes | 97,804 | 57.0 | | 3,421 | | 49.4 | | 2679 | | 49.7 | | 481 | | 54.5 | | 261 | | 39.7 | |
| Abbreviations: CBC, Contralateral breast cases; BCS; breast-conserving surgery; ER,estrogen receptor | | | | | | | | | | | | | | | | | | | |
| ^a^ Among 178,524 ER+ 1^st^ breast cancer patients | | | | | | | | | | | | | | | | | | | |

| Supplemental Table 7 - Relative risk (95%CI) for contralateral breast cancer according to selected patient and clinical characteristics among 5-year breast cancer survivors in SEER cohort. | | | | |  |  |
| --- | --- | --- | --- | --- | --- | --- |
|  | **SEER cohort** | | | |  |  |
|  | **All Contralateral Breast Cases** | | | |  |  |
| **Characteristics of 1^st^ breast cancer patients** | **Observed** | **RR^a^** | **95%CI** | **P-value^b^** |  |  |
| **Overall** | 10470 |  |  |  |  |  |
| **Age at diagnosis, year** |  |  |  |  |  |  |
| 20-39 | 817 | 1 |  |  |  |  |
| 40-54 | 3876 | 0.54 | 0.50-0.58 |  |  |  |
| 55-69 | 4208 | 0.48 | 0.45-0.53 |  |  |  |
| 70-84 | 1,569 | 0.47 | 0.43-0.51 | <0.001 |  |  |
| **Year of diagnosis** |  |  |  |  |  |  |
| 1990-1997 | 5004 | 1 |  |  |  |  |
| 1998-2005 | 4143 | 1.01 | 0.97-1.06 |  |  |  |
| 2006-2013 | 1323 | 0.89 | 0.84-0.95 | <0.001 |  |  |
| **Stage** |  |  |  |  |  |  |
| Localised | 7632 | 1 |  |  |  |  |
| Regional | 2838 | 0.98 | 0.94-1.03 | 0.50 |  |  |
| **Grade^c^** |  |  |  |  |  |  |
| 1 | 1893 | 1 |  |  |  |  |
| 2 | 3567 | 0.93 | 0.88-0.99 |  |  |  |
| 3 | 3133 | 1.04 | 0.98-1.10 |  |  |  |
| 4 | 228 | 1.00 | 0.88-1.17 | <0.001 |  |  |
| **Tumor size, cm^d^** |  |  |  |  |  |  |
| < 1 | 2375 | 1 |  |  |  |  |
| 1-<2 | 4056 | 0.96 | 0.91-1.00 |  |  |  |
| 2-<5 | 3104 | 1.00 | 0.94-1.06 |  |  |  |
| 5+ | 504 | 1.14 | 1.03-1.26 | 0.0028 |  |  |
| **Histology** |  |  |  |  |  |  |
| Ductal | 7694 | 1 |  |  |  |  |
| Lobular | 800 | 1.04 | 0.97-1.34 |  |  |  |
| Mixed | 779 | 1.25 | 1.16-1.34 |  |  |  |
| Others | 1197 | 1.00 | 0.94-1.06 | <0.001 |  |  |
| **ER status^e^** |  |  |  |  |  |  |
| ER+ | 6932 | 1 |  |  |  |  |
| ER- | 2215 | 1.12 | 1.07-1.19 |  |  |  |
| ER unknown | 1323 | 0.97 | 0.92-1.04 | <0.001 |  |  |
|  | **ER+ Contralateral Breast Cases** | | | |  |  |
| Overall | 7590 |  |  |  |  |  |
| **Age at diagnosis, years** |  |  |  |  |  |  |
| 20-39 | 428 | 1 |  |  |  |  |
| 40-54 | 2750 | 0.69 | 0.62-0.76 |  |  |  |
| 55-69 | 3228 | 0.64 | 0.58-0.71 |  |  |  |
| 70-84 | 1,184 | 0.62 | 0.56-0.70 | <0.001 |  |  |
| **Year of diagnosis** |  |  |  |  |  |  |
| 1990-1997 | 3419 | 1 |  |  |  |  |
| 1998-2005 | 3132 | 1.01 | 0.96-1.06 |  |  |  |
| 2006-2013 | 1039 | 0.88 | 0.81-0.94 | <0.001 |  |  |
| **Stage** |  |  |  |  |  |  |
| Localised | 5565 | 1 |  |  |  |  |
| Regional | 2025 | 1.03 | 0.97-1.09 | 0.37 |  |  |
| **Grade^c^** |  |  |  |  |  |  |
| 1 | 1526 | 1 |  |  |  |  |
| 2 | 2746 | 0.92 | 0.86-0.98 |  |  |  |
| 3 | 2018 | 0.89 | 0.83-0.96 |  |  |  |
| 4 | 143 | 0.87 | 0.73-1.03 | 0.0075 |  |  |
| **Tumor size, cm^d^** |  |  |  |  |  |  |
| < 1 | 1815 | 1 |  |  |  |  |
| 1-<2 | 3022 | 0.97 | 0.91-1.03 |  |  |  |
| 2-<5 | 2130 | 0.98 | 0.91-1.05 |  |  |  |
| 5+ | 336 | 1.11 | 0.98-1.25 | 0.13 |  |  |
| **Histology** |  |  |  |  |  |  |
| Ductal | 5528 | 1 |  |  |  |  |
| Lobular | 629 | 1.10 | 1.00-1.20 |  |  |  |
| Mixed | 602 | 1.29 | 1.19-1.41 |  |  |  |
| Others | 831 | 0.98 | 0.91-1.05 | <0.001 |  |  |
| **ER status^e^** |  |  |  |  |  |  |
| ER+ | 5393 | 1 |  |  |  |  |
| ER- | 1274 | 0.90 | 0.84-0.96 |  |  |  |
| ER unknown | 923 | 0.92 | 0.85-0.99 | 0.0013 |  |  |
|  | **ER- Contralateral Breast Cases** | | | |  |  |
| **Overall** | 1838 |  |  |  |  |  |
| **Age at diagnosis, years** |  |  |  |  |  |  |
| 20-39 | 288 | 1 |  |  |  |  |
| 40-54 | 756 | 0.37 | 0.32-0.42 |  |  |  |
| 55-69 | 595 | 0.30 | 0.26-0.35 |  |  |  |
| 70-84 | 199 | 0.31 | 0.25-0.37 | <0.001 |  |  |
| **Year of diagnosis** |  |  |  |  |  |  |
| 1990-1997 | 857 | 1 |  |  |  |  |
| 1998-2005 | 751 | 1.05 | 0.94-1.16 |  |  |  |
| 2006-2013 | 230 | 0.98 | 0.84-1.14 | 0.24 |  |  |
| **Stage** |  |  |  |  |  |  |
| Localised | 1297 | 1 |  |  |  |  |
| Regional | 541 | 0.85 | 0.76-0.95 | 0.0046 |  |  |
| **Grade^c^** |  |  |  |  |  |  |
| 1 | 203 | 1 |  |  |  |  |
| 2 | 478 | 1.08 | 0.91-1.27 |  |  |  |
| 3 | 839 | 2.04 | 1.73-2.40 |  |  |  |
| 4 | 55 | 1.90 | 1.41-2.57 | <0.001 |  |  |
| **Tumor size, cm^d^** |  |  |  |  |  |  |
| < 1 | 329 | 1 |  |  |  |  |
| 1-<2 | 642 | 0.94 | 0.82-1.08 |  |  |  |
| 2-<5 | 675 | 1.09 | 0.94-1.26 |  |  |  |
| 5+ | 112 | 1.16 | 0.92-1.45 | 0.029 |  |  |
| **Histology** |  |  |  |  |  |  |
| Ductal | 1417 | 1 |  |  |  |  |
| Lobular | 101 | 0.87 | 0.70-1.07 |  |  |  |
| Mixed | 88 | 0.85 | 0.68-1.06 |  |  |  |
| Others | 232 | 1.09 | 0.94-1.25 | 0.13 |  |  |
| **ER status^e^** |  |  |  |  |  |  |
| ER+ | 882 | 1 |  |  |  |  |
| ER- | 729 | 2.16 | 1.94-2.41 |  |  |  |
| ER unknown | 227 | 1.27 | 1.09-1.47 | <0.001 |  |  |
| Abbreviations: RR, relative risk; CI, confidence interval; ER, estrogen receptor | | | | |  |  |
| ^a^ Relative risk estimated using Poisson regression, adjusted for year and age at diagnosis, radiotherapy, chemotherapy, hormone therapy, grade and tumor size as appropriate.  ^b^ Likelihood ratio test for heterogeneity of relative risks. | | | | |  |  |
| ^c^ Excluded patients with unknown grade (N=28,941)  ^d^ Excluded patients with unknown tumour size (N=8,183)  ^e^ Model did not include adjustment for hormone therapy | | | | |  |  |

| Supplemental Table 8 - Relative risk (95%CI) for contralateral breast cancer and radiotherapy stratified by age at first breast cancer diagnosis among 5-year breast cancer survivors in the SEER cohort. | | | | | | | | | | | | | | | | | | | |
| --- | --- | --- | --- | --- | --- | --- | --- | --- | --- | --- | --- | --- | --- | --- | --- | --- | --- | --- | --- |
|  | **Radiotherapy (All patients)** | | | | |  | **Radiotherapy with hormone therapy (ER+ 1st breast cancer only)** | | | | |  | **Radiotherapy without hormone therapy^a^ (ER+ 1st breast cancer only)** | | | | | |  |
|  | **All Contralateral Breast Cases** | | | | | | | | | | | | | | | | | |  |
| Age at 1st breast cancer diagnosis | No/  Unknown | Yes |  |  |  |  | No/  Unknown | Yes |  |  |  |  | No/  Unknown | Yes |  |  | |  |  |
|  | Obs | Obs | RR | 95%CI | P^b^ |  | Obs | Obs | RR | 95%CI | P^b^ |  | Obs | Obs | RR | 95%CI | | P^b^ |  |
| 20-39 | 442 | 375 | 1.15 | 0.99-1.34 |  |  | 102 | 54 | 0.87 | 0.61-1.22 |  |  | 114 | 99 | 1.45 | 1.09-1.93 | |  |  |
| 40-54 | 2227 | 1649 | 1.07 | 1.00-1.15 |  |  | 833 | 329 | 1.03 | 0.90-1.17 |  |  | 638 | 618 | 1.09 | 0.97-1.22 | |  |  |
| 55-69 | 2430 | 1778 | 1.05 | 0.99-1.13 |  |  | 1127 | 450 | 1.02 | 0.91-1.15 |  |  | 708 | 702 | 1.09 | 0.98-1.22 | |  |  |
| 70+ | 772 | 797 | 1.02 | 0.91-1.13 | 0.46 |  | 338 | 188 | 1.07 | 0.89-1.29 | 0.83 |  | 267 | 365 | 1.01 | 0.85-1.19 | | 0.12 |  |
|  | **ER+ Contralateral Breast Cases** | | | | | | | | | | | | | | | | | |  |
| 20-39 | 235 | 193 | 1.11 | 0.90-1.36 |  |  | 79 | 39 | 0.94 | 0.24-3.68 |  |  | 77 | 67 | 1.37 | 0.97-1.95 | |  |  |
| 40-54 | 1588 | 1162 | 1.05 | 0.97-1.14 |  |  | 649 | 258 | 1.02 | 0.88-1.18 |  |  | 493 | 476 | 1.09 | 0.95-1.24 | |  |  |
| 55-69 | 1901 | 1327 | 1.07 | 0.99-1.15 |  |  | 902 | 358 | 0.99 | 0.87-1.13 |  |  | 570 | 542 | 1.13 | 1.00-1.27 | |  |  |
| 70+ | 321 | 563 | 1.12 | 0.99-1.26 | 0.62 |  | 264 | 130 | 1.18 | 0.95-1.46 | 0.51 |  | 226 | 263 | 1.13 | 0.94-1.36 | | 0.67 |  |
|  | **ER- Contralateral Breast Cases** | | | | | | | | | | | | | | | | | |  |
| 20-39 | 158 | 130 | 1.31 | 1.02-1.69 |  |  | 12 | 5 | 1.10 | 0.38-3.22 |  |  | 23 | 16 | **2.15** | **1.09-4.23** | |  |  |
| 40-54 | 448 | 308 | 1.19 | 1.02-1.40 |  |  | 122 | 49 | 0.95 | 0.67-1.34 |  |  | 81 | 65 | 1.35 | 0.96-1.89 | |  |  |
| 55-69 | 352 | 243 | 1.07 | 0.89-1.27 |  |  | 162 | 52 | 1.23 | 0.89-1.71 |  |  | 78 | 79 | 0.98 | 0.71-1.36 | |  |  |
| 70+ | 81 | 118 | 0.66 | 0.49-0.90 | **0.019** |  | 44 | 35 | 0.73 | 0.46-1.16 | 0.28 |  | 16 | 43 | 0.52 | 0.29-0.94 | | 0.0077 |  |
| Abbreviations: Obs, observed number of cases; RR, relative risk; CI, confidence interval; ER, estrogen receptor | | | | | | | | | | | | | |  |  |  | |  |  |
| ^a^ Patients who received radiotherapy and no/unknown therapy receipt  ^b^ Likelihood ratio test for heterogeneity of relative risks. | | | | | | | | | | | | | | | | |  | |  |

| Supplemental Table 9 -Selected patient and clinical characteristics by radiotherapy status among non-cases in the SEER cohort. | | | | | | | | | | | | |
| --- | --- | --- | --- | --- | --- | --- | --- | --- | --- | --- | --- | --- |
|  | **SEER cohort** | | | | | | | | | | |  |
|  | **No/Unknown RT** | | | **RT** | | | | **Total** | | | |  |
| **Characteristics of 1^st^ breast cancer patients** | **N** | **%** | | **N** | | **%** | | **N** | | **%** | |  |
| Overall | 98,230 | 41.9 | | 136,134 | | 58.1 | | 234,364 | | 100.0 | |  |
| Age at diagnosis, years |  |  | |  | |  | |  | |  | |  |
| 20-39 | 5,782 | 44.0 | | 7,345 | | 56.0 | | 13,127 | | 100.0 | |  |
| 40-54 | 30,022 | 39.2 | | 46,511 | | 60.8 | | 76,533 | | 100.0 | |  |
| 55-69 | 33,985 | 38.5 | | 54,225 | | 61.5 | | 88,210 | | 100.0 | |  |
| 70-84 | 28,441 | 50.3 | | 28,053 | | 49.7 | | 56,494 | | 100.0 | |  |
| Age at diagnosis (ER+ only), years | | |  | |  | |  | |  | |  | |
| 20-39 | 3,146 | 41.0 | | 4,525 | | 59.0 | | 7,671 | | 100.0 | |  |
| 40-54 | 19,358 | 35.9 | | 34,491 | | 64.1 | | 53,849 | | 100.0 | |  |
| 55-69 | 23,132 | 34.9 | | 43,168 | | 65.1 | | 66,300 | | 100.0 | |  |
| 70-84 | 20,731 | 47.4 | | 23,041 | | 52.6 | | 43,772 | | 100.0 | |  |
| Year of diagnosis |  |  | |  | |  | |  | |  | |  |
| 1990-1993 | 21,158 | 63.1 | | 12,388 | | 36.9 | | 33,546 | | 100.0 | |  |
| 1994-1997 | 18,835 | 50.6 | | 18,373 | | 49.4 | | 37,208 | | 100.0 | |  |
| 1998-2001 | 17,458 | 41.6 | | 24,530 | | 58.4 | | 41,988 | | 100.0 | |  |
| 2002-2005 | 14,453 | 36.2 | | 25,522 | | 63.8 | | 39,975 | | 100.0 | |  |
| 2006-2009 | 14,135 | 34.8 | | 26,452 | | 65.2 | | 40,587 | | 100.0 | |  |
| 2010-2013 | 12,191 | 29.7 | | 28,869 | | 70.3 | | 41,060 | | 100.0 | |  |
| Stage |  |  | |  | |  | |  | |  | |  |
| Localised | 70,413 | 41.8 | | 98,104 | | 58.2 | | 168517 | | 100.0 | |  |
| Regional | 27,817 | 42.2 | | 38,030 | | 57.8 | | 65847 | | 100.0 | |  |
| Grade |  |  | |  | |  | |  | |  | |  |
| 1 | 16,369 | 34.5 | | 31,145 | | 65.5 | | 47,514 | | 100.0 | |  |
| 2 | 35,779 | 39.6 | | 54,576 | | 60.4 | | 90,355 | | 100.0 | |  |
| 3 | 28,134 | 42.7 | | 37,711 | | 57.3 | | 65,845 | | 100.0 | |  |
| 4 | 1,793 | 53.4 | | 1,565 | | 46.6 | | 3,358 | | 100.0 | |  |
| Unknown | 16,155 | 59.2 | | 11,137 | | 40.8 | | 27,292 | | 100.0 | |  |
| Tumor size, cm |  |  | |  | |  | |  | |  | |  |
| < 1 | 20,039 | 37.2 | | 33,823 | | 62.8 | | 53,862 | | 100.0 | |  |
| 1-<2 | 35,456 | 38.8 | | 55,973 | | 61.2 | | 91,429 | | 100.0 | |  |
| 2-<5 | 33,650 | 47.8 | | 36,800 | | 52.2 | | 70,450 | | 100.0 | |  |
| 5+ | 4,295 | 39.5 | | 6,586 | | 60.5 | | 10,881 | | 100.0 | |  |
| Unknown | 4,790 | 61.8 | | 2,962 | | 38.2 | | 7,752 | | 100.0 | |  |
| ER status |  |  | |  | |  | |  | |  | |  |
| ER+ | 68,192 | 38.7 | | 108,117 | | 61.3 | | 176,309 | | 100.0 | |  |
| ER- | 15,588 | 44.4 | | 19,525 | | 55.6 | | 35,113 | | 100.0 | |  |
| ER unknown | 14,450 | 63.0 | | 8,492 | | 37.0 | | 22,942 | | 100.0 | |  |
| Surgery type |  |  | |  | |  | |  | |  | |  |
| BCS | 24,039 | 16.9 | | 118,260 | | 83.1 | | 142,299 | | 100.0 | |  |
| Mastectomy | 74,131 | 80.6 | | 17,830 | | 19.4 | | 91,961 | | 100.0 | |  |
| Surgery, NOS | 60 | 57.7 | | 44 | | 42.3 | | 104 | | 100.0 | |  |
| Chemotherapy |  |  | |  | |  | |  | |  | |  |
| No/Unknown | 69,562 | 46.6 | | 79,723 | | 53.4 | | 149,285 | | 100.0 | |  |
| Yes | 28,668 | 33.7 | | 56,411 | | 66.3 | | 85,079 | | 100.0 | |  |
| Abbreviations: ER, Estrogen receptor; RT, Radiotherapy; BCS, breast-conserving surgery; NOS, Not specified; | | | | | | | | | | | | |

| Supplemental Table 10 - Selected patient and clinical characteristics by radiotherapy status and hormone therapy receipt - Restricted to ER+ non-cases patients. | | | | | | | | | | | | | |
| --- | --- | --- | --- | --- | --- | --- | --- | --- | --- | --- | --- | --- | --- |
|  | **SEER cohort** | | | | | | | | | | | | |
|  | **No/Unknown Hormone therapy** | | | | | |  | **Hormone therapy** | | | | | |
|  | **No/Unknown RT** | | **RT** | | **Total** | |  | **No/Unknown RT** | | **RT** | | **Total** | |
| **Characteristics of 1^st^ breast cancer patients** | **N** | **%** | **N** | **%** | **N** | **%** |  | **N** | **%** | **N** | **%** | **N** | **%** |
| **Overall** | 39700 | 53.8 | 34088 | 46.2 | 73788 | 100.0 |  | 26667 | 27.3 | 71137 | 72.7 | 97804 | 100.0 |
| **Age diagnosis, years** | |  |  |  |  |  |  |  |  |  |  |  |  |
| 20-39 | 2060 | 54.4 | 1727 | 45.6 | 3787 | 100.0 |  | 1086 | 28.0 | 2798 | 72.0 | 3884 | 100.0 |
| 40-54 | 11842 | 51.3 | 11237 | 48.7 | 23079 | 100.0 |  | 7516 | 24.4 | 23254 | 75.6 | 30770 | 100.0 |
| 55-84 | 25798 | 55.0 | 21124 | 45.0 | 46922 | 100.0 |  | 18065 | 28.6 | 45085 | 71.4 | 63150 | 100.0 |
| **Year of diagnosis** |  |  |  |  |  |  |  |  |  |  |  |  |  |
| 1990-1993 | 7922 | 65.8 | 4109 | 34.2 | 12031 | 100.0 |  | 4531 | 54.1 | 3837 | 45.9 | 8368 | 100.0 |
| 1994-1997 | 7754 | 54.6 | 6449 | 45.4 | 14203 | 100.0 |  | 3866 | 37.7 | 6398 | 62.3 | 10264 | 100.0 |
| 1998-2001 | 7400 | 51.2 | 7042 | 48.8 | 14442 | 100.0 |  | 4204 | 27.3 | 11219 | 72.7 | 15423 | 100.0 |
| 2002-2005 | 6360 | 48.3 | 6805 | 51.7 | 13165 | 100.0 |  | 3750 | 22.7 | 12769 | 77.3 | 16519 | 100.0 |
| 2006-2009 | 5959 | 52.1 | 5480 | 47.9 | 11439 | 100.0 |  | 4717 | 22.4 | 16344 | 77.6 | 21061 | 100.0 |
| 2010-2013 | 4305 | 50.6 | 4203 | 49.4 | 8508 | 100.0 |  | 5599 | 21.4 | 20570 | 78.6 | 26169 | 100.0 |
| **Stage** |  |  |  |  |  |  |  |  |  |  |  |  |  |
| Localized | 28286 | 52.2 | 25933 | 47.8 | 54219 | 100.0 |  | 18485 | 27.0 | 50083 | 73.0 | 68568 | 100.0 |
| Regional | 11414 | 58.3 | 8155 | 41.7 | 19569 | 100.0 |  | 8182 | 28.0 | 21054 | 72.0 | 29236 | 100.0 |
| **Grade** |  |  |  |  |  |  |  |  |  |  |  |  |  |
| 1 | 11064 | 48.5 | 11760 | 51.5 | 22824 | 100.0 |  | 5,990 | 22.5 | 20,593 | 77.5 | 26583 | 100.0 |
| 2 | 24049 | 53.4 | 20980 | 46.6 | 45029 | 100.0 |  | 13,245 | 27.1 | 35,648 | 72.9 | 48893 | 100.0 |
| 3 | 22354 | 49.5 | 22837 | 50.5 | 45191 | 100.0 |  | 7,123 | 29.9 | 16,664 | 70.1 | 23787 | 100.0 |
| 4 | 1311 | 57.6 | 964 | 42.4 | 2275 | 100.0 |  | 589 | 44.9 | 722 | 55.1 | 1311 | 100.0 |
| Unknown | 12816 | 65.7 | 6700 | 34.3 | 19516 | 100.0 |  | 4,288 | 45.5 | 5,137 | 54.5 | 9425 | 100.0 |
| **Tumor size, cm** |  |  |  |  |  |  |  |  |  |  |  |  |  |
| < 1 | 15458 | 47.8 | 16877 | 52.2 | 32335 | 100.0 |  | 5,535 | 23.2 | 18,367 | 76.8 | 23902 | 100.0 |
| 1-<2 | 24842 | 50.7 | 24146 | 49.3 | 48988 | 100.0 |  | 12,206 | 26.3 | 34,291 | 73.7 | 46497 | 100.0 |
| 2-<5 | 23932 | 58.0 | 17339 | 42.0 | 41271 | 100.0 |  | 11,286 | 35.0 | 20,987 | 65.0 | 32273 | 100.0 |
| 5+ | 3309 | 52.2 | 3027 | 47.8 | 6336 | 100.0 |  | 1,212 | 24.0 | 3,837 | 76.0 | 5049 | 100.0 |
| Unknown | 4053 | 68.6 | 1852 | 31.4 | 5905 | 100.0 |  | 996 | 43.7 | 1,282 | 56.3 | 2278 | 100.0 |
| **Surgery type** |  |  |  |  |  |  |  |  |  |  |  |  |  |
| BCS | 11970 | 28.3 | 30387 | 71.7 | 42357 | 100.0 |  | 3975 | 6.0 | 61787 | 94.0 | 65762 | 100.0 |
| Mastectomy | 27716 | 88.2 | 3692 | 11.8 | 31408 | 100.0 |  | 22685 | 70.8 | 9334 | 29.2 | 32019 | 100.0 |
| NOS | 14 | 60.9 | 9 | 39.1 | 23 | 100.0 |  | 7 | 30.4 | 16 | 69.6 | 23 | 100.0 |
| **Chemotherapy** |  |  |  |  |  |  |  |  |  |  |  |  |  |
| No/Unknown | 30351 | 57.1 | 22786 | 42.9 | 53137 | 100.0 |  | 18032 | 28.9 | 44448 | 71.1 | 62480 | 100.0 |
| Yes | 9349 | 45.3 | 11302 | 54.7 | 20651 | 100.0 |  | 8635 | 24.4 | 26689 | 75.6 | 35324 | 100.0 |
| Abbreviations: ER, estrogen receptor; RT, radiotherapy; BCS, breast-conserving surgery, NOS, Not specified. | | | | | | | | | | | | | |
